# Cardio-omentopexy requires a cardioprotective innate immune response to promote myocardial angiogenesis in mice

**DOI:** 10.1016/j.xjon.2022.02.027

**Published:** 2022-02-24

**Authors:** Zhi-Dong Ge, Riley M. Boyd, Connor Lantz, Edward B. Thorp, Joseph M. Forbess

**Affiliations:** a The Heart Center and Cardiovascular-Thoracic Surgery, Stanley Manne Children's Research Institute, Ann & Robert H. Lurie Children's Hospital of Chicago, Feinberg School of Medicine, Northwestern University, Chicago, Ill; b Department of Surgery, University of Maryland School of Medicine and The Children's Heart Program, University of Maryland Children's Hospital, Baltimore, Md

**Keywords:** cardio-omentopexy, angiogenesis, cardiac hypertrophy, macrophages, Akt, protein kinase B, AXL, AXL receptor tyrosine kinase, Calm1, calmodulin 1, CD45, lymphocyte common antigen, CD64, cluster of differentiation 64, Cdh5, cadherin 5, Clodro, clodronate-liposomes, COP, cardio-omentopexy, Crk, proto-oncogene c-Crk, Ctnnb1, catenin β1, Ctnnd1, catenin delta 1, Cybb, cytochrome B-245 beta chain, Cyfip1, cytoplasmic FMR1 interacting protein 1, ECM, extracellular matrix, F4/80, F4/80 antigen, HCM, hypertrophic cardiomyopathy, Hippo, hippocampal, HSP89aa1, heat shock protein 89aa1, iB4, biotinylated-isolectin B4, Itpr2, inositol 1,4,5-trisphosphate receptor type 2, Kras, kirsten rat sarcoma virus, Kdr, kinase insert domain receptor, LV, left ventricle, Ly6C^lo^, lymphocyte antigen-6C^low^, Ly6G, lymphocyte antigen 6 complex locus G6D, Lyve1, lymphatic vessel endothelial hyaluronan receptor 1, MHCII^lo^, major histocompatibility complex class II^low^, *mTOR*, mammalian target of rapamycin, Ncf1, neutrophil cytosolic factor 1, Nck2, NCK adaptor protein 2, Nckap1H, NCK-associated protein 1H, Nos3, nitric oxide synthase 3, PBS, phosphate-buffered saline, PDGF, platelet-derived growth factor, *PI3K*, phosphoinositide-3-kinase, Plcg1, phospholipase Cγ1, Plcg2, 1-phosphatidylinositol-4,5-bisphosphate phosphodiesterase γ2, Prkaca, protein kinase cAMP-activated catalytic subunit α, Prkca, protein kinase Cα, Prkacb, protein kinase cAMP-activated catalytic subunit β, Ptk2, protein tyrosine kinase 2, Ptk2b, protein tyrosine kinase 2β, Rac1, Rac family small GTPase 1, Rock2, Rho associated coiled-coil containing protein kinase 2, Src, proto-oncogene tyrosine-protein kinase Src, TAC, transverse aortic constriction, TGF, transforming growth factor, TimD4, T cell immunoglobulin and mucin domain conataining 4, Vav1, Vav guanine nucleotide exchange factor 1, VEGF-A, vascular endothelial growth factor A, WGA, wheat germ agglutinin

## Abstract

**Objective:**

The pedicled greater omentum, when applied onto stressed hearts using omentopexy, has been shown to be protective in humans and animals. The mechanisms underlying cardioprotection using omentopexy remain elusive. This study examined whether macrophage-mediated angiogenesis accounts for the cardioprotective effect of omentopexy in mice.

**Methods:**

C57BL/6 mice were subjected to minimally invasive transverse aortic constriction for 6 weeks and subsequent cardio-omentopexy for 8 weeks. Control mice underwent the same surgical procedures without aortic constriction or cardio-omentopexy.

**Results:**

Transverse aortic constriction led to left ventricular concentric hypertrophy, reduced mitral E/A ratio, increased cardiomyocyte size, and myocardial fibrosis in the mice that underwent sham cardio-omentopexy surgery. The negative effects of transverse aortic constriction were prevented by cardio-omentopexy. Myocardial microvessel density was elevated in the mice that underwent aortic constriction and sham cardio-omentopexy surgery, and cardio-omentopexy further enhanced angiogenesis. Nanostring gene array analysis uncovered the activation of angiogenesis gene networks by cardio-omentopexy. Flow cytometric analysis revealed that cardio-omentopexy triggered the accumulation of cardiac MHCII^lo^Lyve1+TimD4+ (Major histocompatibility complex class II^low^ lymphatic vessel endothelial hyaluronan receptor 1+ T cell immunoglobulin and mucin domain conataining 4+) resident macrophages at the omental–cardiac interface. Intriguingly, the depletion of macrophages with clodronate-liposome resulted in the failure of cardio-omentopexy to protect the heart and promote angiogenesis.

**Conclusions:**

Cardio-omentopexy protects the heart from pressure overload-elicited left ventricular hypertrophy and dysfunction by promoting myocardial angiogenesis. Cardiac MHCII^lo^Lyve1+TimD4+ resident macrophages play a critical role in the cardioprotective effect and angiogenesis of cardio-omentopexy.

**Video Abstract:**

## Graphical abstract

**Figure:**
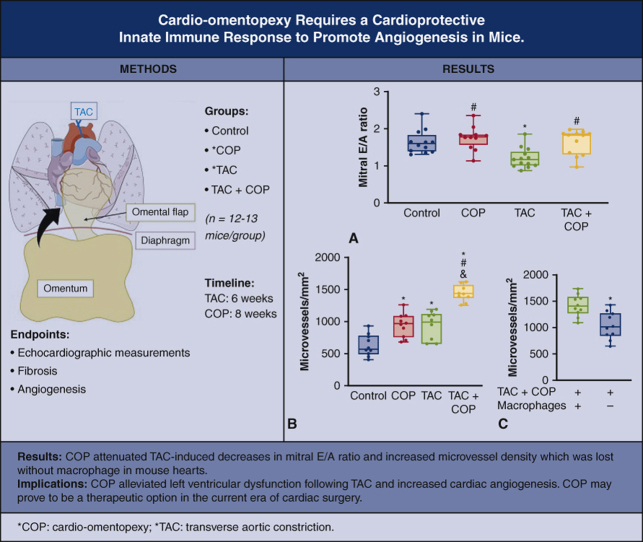

**Figure:**
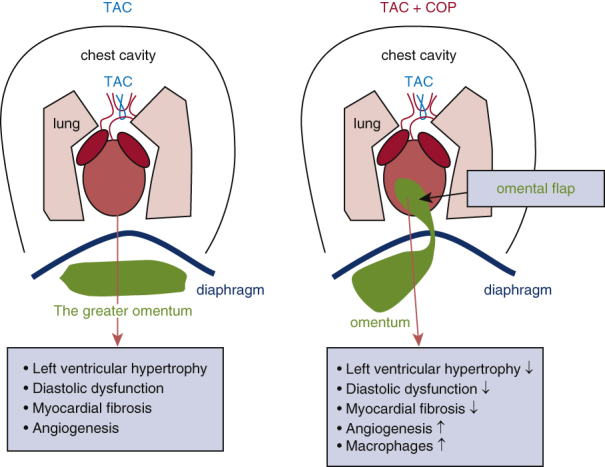
Cardio-omentopexy ameliorates TAC-induced diastolic dysfunction via angiogenesis in mice.

**Central Message**

Cardio-omentopexy protects the heart from pressure overload-induced left ventricular hypertrophy and dysfunction through myocardial angiogenesis and MHCII^lo^Lyve1+TimD4+ resident cardiac macrophages in mice.

**Perspective**

Although cardio-omentopexy benefits ischemic hearts, it has been supplanted by coronary artery bypass grafting. The authors present that cardio-omentopexy activates a pro-reparative inflammatory response accompanied by cardiac angiogenesis and reductions in cardiac hypertrophy and cardiomyocyte cell size, with macrophages as central protagonists. In this work the therapeutic benefit and potential mechanisms of cardio-omentopexy in ameliorating pressure overload-elicited cardiac hypertrophy and dysfunction in mice are presented.

The greater omentum is an apron-like fold with a rich vascular supply in the visceral peritoneum. It has been noted that the human omentum can promote local angiogenic activity. The greater omentum contains leukocyte aggregates, called milky spots, that are rich in macrophages and support the innate and adaptive immune response to peritoneal antigens. Separate studies have shown the greater omentum to also be a source of pluripotent stem cells and growth factors.^1^, ^2^, ^3^

The surgical procedure of omentopexy, in which the greater omentum is sutured to an organ, has long been known to harbor therapeutic and vascularizing potential.^4^, ^5^, ^6^ In the case of heart, “cardio-omentopexy” (COP) increases collateral circulation and was first coined in the 1930s by Laurence O'Shaughnessy^7^ to describe a procedure developed to treat angina secondary to coronary artery disease. O'Shaughnessy reported that using COP in greyhounds with ligated left coronary arteries resulted in vascular anastomoses and that these animals sustained their racing conditioning. Despite its subsequent reproductibility and efficacy,^8^ COP was superceded by another surgical procedure, coronary artery bypass grafting, which provides the patients with myocardial infarction with higher survival rates, better left ventricular function, and a decrease in rates of heart failure.^9^

Recently, a study from our laboratory showed that COP ameliorated left ventricular hypertrophy and dysfunction in a rat model of pressure overload.^10^ The underlying mechanisms of action remain unclear. This investigation first confirms that COP has angiogenic and cardioprotective roles in a murine model of pressure overload (Video 1). On the basis of the known immunological properties of the greater omentum, we hypothesized that COP protects the heart against pressure overload-induced cardiac hypertrophy and dysfunction via a macrophage-dependent mechanism of myocardial angiogenesis.

## Methods

### Animals

The general structure of the greater omentum in adult mice is similar to that in human fetus and in children.^11^ We used C57BL/6 mice from the Jackson Laboratory in the current study. Male C57BL/6 mice (20-25 g; 8-10 weeks of age) were used and bred and cared for in the Animal Facility of Northwestern University Chicago Campus. Animal care and all experimental procedures were performed in accordance with the National Institutes of Health *Guide for the Care and Use of Laboratory Animals* (8th edition, 2011), and experimental protocols were approved by the Institutional Animal Care and Use Committee at Northwestern University (protocol number: IS000010625; date of approval: January 10, 2019). Expanded methods are described in the Online Data Supplement.

### Experimental Outline

**Figure 1:**
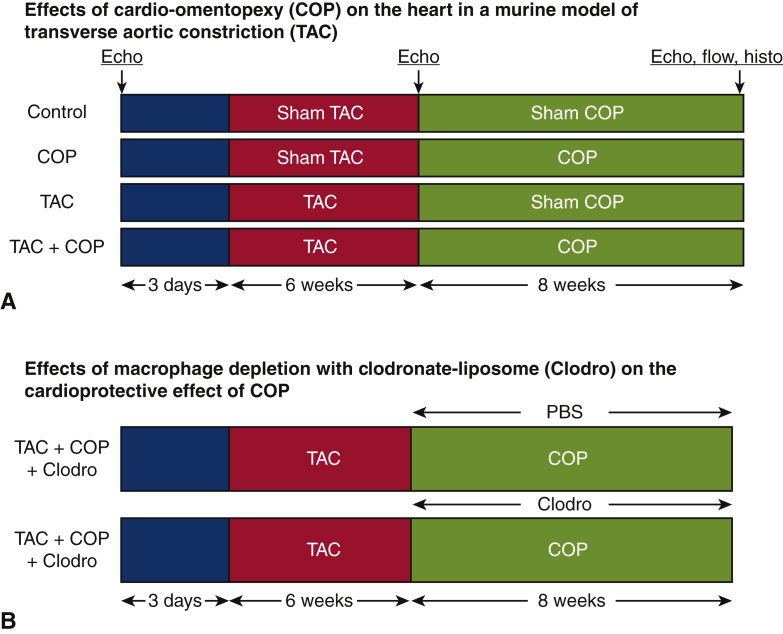
Experimental protocol. A, Effects of cardio-omentopexy (COP) on the heart in a murine model of transverse aortic constriction (TAC). In the TAC+COP group, C57BL/6 mice were subjected to TAC for 6 weeks and subsequently COP for 8 weeks. The transverse aorta was constricted between the innominate and left common carotid arteries using a 7-0 prolene suture ligature tied against a 25-gauge blunted needle. The pedicled greater omentum with the right gastroepiploic artery was transferred to the heart through the diaphragm. Mice in TAC and COP groups underwent TAC or COP alone. Control mice underwent all surgical procedures without the TAC and/or connection of the greater omentum with the heart. B, Effects of macrophage depletion with clodronate-liposome (*Clodro*) on the cardioprotective effect of COP. The geometry and function of the left ventricle was evaluated with echocardiography (*Echo*). Flow cytometry (*flow*) was conducted to analyze macrophage subsets in mouse hearts, and mouse hearts received histopathological examination (*histo*) to determine cardiomyocyte size, fibrosis, and microvessel density.

The experimental protocol is outlined in Figure 1. The effects of COP on pressure overload-induced hypertrophy and cardiac dysfunction were determined in C57BL/6 mice randomly assigned to 4 experimental groups: control (n = 12 mice), COP (n = 12 mice), transverse aortic constriction (TAC; n = 13 mice), and TAC+COP (n = 12 mice) (Figure 1, *A*). Control mice were subjected to sham TAC surgery for 6 weeks and subsequent sham COP surgery for 8 weeks. The mice in the COP group were subjected to sham TAC surgery for 6 weeks and subsequent COP for 8 weeks. The animals in the TAC group underwent TAC for 6 weeks and subsequent sham COP surgery for 8 weeks. In the TAC+COP group, the mice were subjected to TAC for 6 weeks and subsequent COP for 8 weeks. The morphology and function of the left ventricle (LV) at baseline (3 days before TAC or sham TAC surgery), 6 weeks post TAC, and 8 weeks post COP were evaluated using echocardiography. Myocardial fibrosis, cardiomyocyte size, microvessel density, and cardiac macrophages were quantified at 8 weeks after COP or sham surgery. The role of cardiac macrophages in the cardioprotective effect of COP was examined in COP+TAC mice through depletion of macrophages with clodronate-liposomes (Clodro; Figure 1, *B*).

### TAC

**Figure E1:**
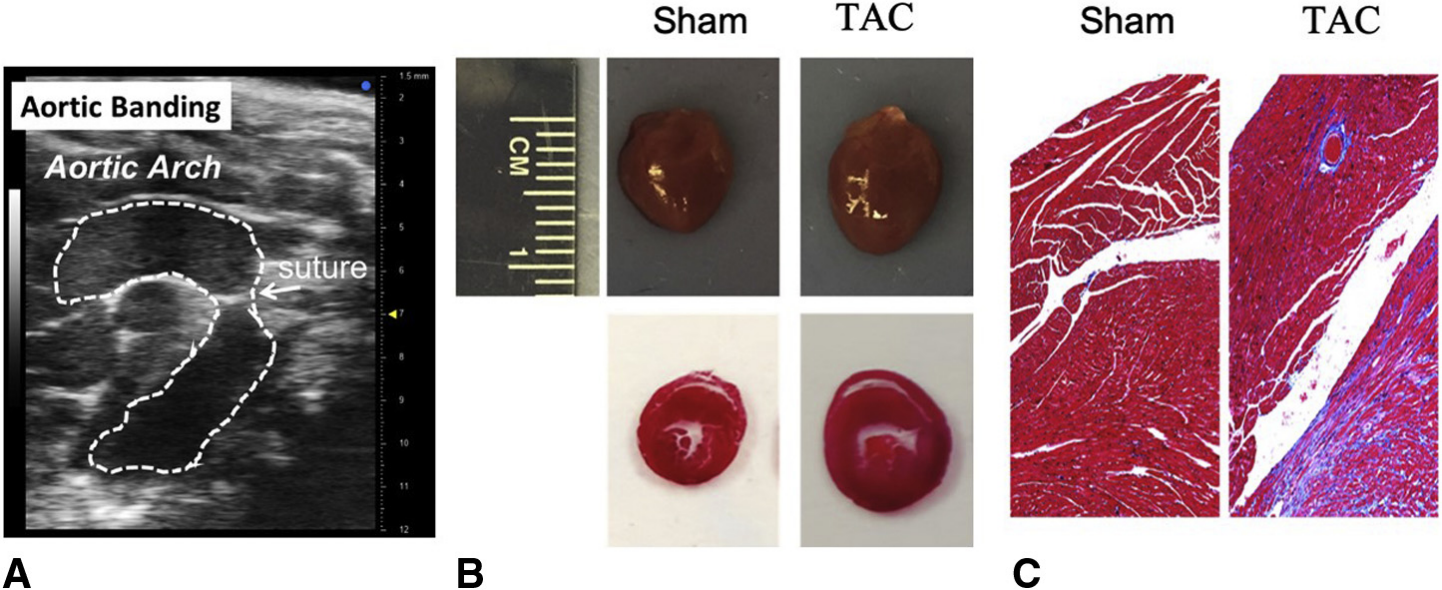
Validation of mouse model of cardiac hypertrophy using transverse aortic constriction (*TAC*) according to gross pathology and fibrosis. The aortic arch of C57BL/6 mice was constricted between the innominate artery and left common carotid artery for 6 weeks. Sham mice underwent all surgical procedures except constriction of the aorta. A, Echocardiographic image showing aortic banding in a mouse; (B) validation of TAC-induced cardiac hypertrophy; and (C) Masson's trichrome-stained heart sections showing fibrosis elicited by TAC.

**Figure E2:**
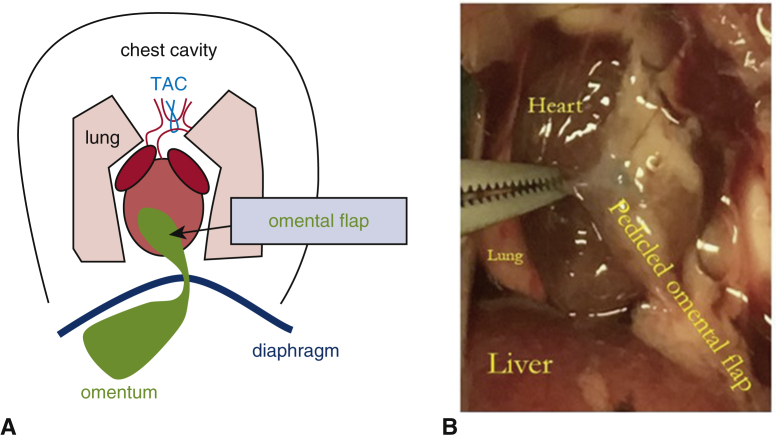
Transverse aortic constriction (*TAC*) and cardio-omentopexy. A, Schematic of the cardio-omentopexy procedure after TAC; and (B) the photograph of cardio-omentopexy in a mouse.

Minimally invasive TAC was performed on male C57BL/6 mice (n = 25; 13 mice in the TAC and 12 mice in the TAC+COP group) at 8 to 10 weeks of age, as described.^12^ Using anesthesia of 1.5% to 2.0% isoflurane, a 0.5-cm horizontal incision was made at the level of the suprasternal notch. The chest was opened 2 to 3 cm in the proximal portion of the sternum. The thymus was deflected to expose the aortic arch. The transverse aorta was constricted between the innominate and left common carotid arteries using a 7-0 prolene suture ligature tied against a 25-gauge blunted needle (Figures E1 and E2). The latter was quickly removed to yield a constriction of 0.4 mm in diameter, which was measured using a VisualSonics Vevo 3100 High-resolution Imaging System.^13^ Sham control mice (n = 24; 12 mice in the control group and 12 mice in the TAC group) underwent all surgical procedures without aortic constriction.

### Murine COP

Six weeks after TAC or sham TAC surgery, male C57BL/6 mice (n = 24; 12 mice in the COP and 12 mice in the TAC+COP group) were initially anesthetized with isoflurane (approximately 4%). When consciousness was lost, the trachea was cannulated with a 25-gauge flexible catheter. The catheter was connected to a mechanical ventilator that provided positive-pressure ventilation. The ventilator was connected to a vaporizer delivering approximately 1.5% isoflurane. After shaving, the surgical areas were scrubbed and disinfected, as previously described.^14^ To prepare the pedicled omental flap, a transverse 1-cm skin incision was made in the left upper quadrant of the abdomen and laparotomy was performed.^15^ One flap with the right gastroepiploic artery was made and translocated into the chest through the diaphragm. A left thoracotomy was performed between the fourth and fifth ribs, and the lungs were retracted to expose the heart.^16^ The pedicled omental flap was sutured to the LV anterior wall with a 7-0 prolene suture (Figure E2). Sham-operated animals (n = 25; 12 mice in the control and 13 mice in the TAC group) underwent the same procedure except for the suturing of the omental flap.

### Transthoracic Echocardiography

Noninvasive transthoracic echocardiography was used to evaluate left ventricular geometry and function in mice at baseline (3 days before TAC or sham TAC surgery), 6 weeks post TAC, and 8 weeks post COP using 1.5% isoflurane. Echocardiography was performed with a VisualSonics Vevo 3100 High-resolution Imaging System.^17^

### Histopathological Examination of Mouse Hearts

To validate the findings from echocardiographic examination of mice, mice at 8 weeks after COP or sham COP surgery were euthanized, and mouse hearts were visualized and weighed. Dehydrated mouse hearts were embedded with paraffin and sliced transversely from the apex to the basal part of the LV at 4- to 5-μm thickness.^13^ Sections were stained with Masson's trichrome to assess myocardial fibrosis, wheat germ agglutinin (WGA) for cardiomyocyte surface area, biotinylated-isolectin B4 (iB4) and CD31 for quantification of myocardial microvessels, or CD68 for visualization of macrophages (n = 10 sections per mouse; 3 mice per group).

#### Masson's trichrome staining

Mice anesthetized with 2,2,2-tribromoethanol received intracardiac saturated KCl (30 mmol/L and 5% dextrose in 1 × phosphate-buffered saline [PBS]) to arrest the heart in diastole, as described.^18^ Mouse hearts were washed with cold PBS, fixed with 10% formalin, dehydrated, and embedded in paraffin. The percentage of total fibrosis area was calculated as the summed, blue-stained regions of interest divided by total area.

#### Immunohistochemical staining

Cardiomyocyte size and myocardial microvessel density were measured by staining mouse hearts with WGA and iB4, respectively.^19^ For measurements of cardiomyocyte size, tissue sections were stained with fluorescence Oregon Green 488-labeled WGA. For the determination of myocardial microvessel density, the slide was boiled to 95 °C in 0.01 mol/L sodium citrate solution and stained with iB4 overnight.

#### Immunofluorescent staining

Immunofluorescent staining with CD31 and CD68 antibodies was used to detect vascular endothelial cells and macrophages. Hearts were embedded in optimum cutting temperature freezing medium; 10-mm transverse sections were cut using a cryostat. Primary antibodies against CD31 and CD68 antigens were added and incubated at 37 °C. Appropriate secondary antibodies were then added and incubated at 37 °C.

### Nanostring Gene Array

Myocardium from TAC (n = 3) and TAC followed by COP (TAC+COP) (n = 3) mice were dissected and stored in RNAlater. Total RNA was extracted with TRIzol reagent following the manufacturer's protocol. Intact RNA (50 ng) was used for Nanostring analysis with the nCounter Fibrosis Panel-Mouse (nanoString). nSolver 4.0 (nanoString) was used for raw count normalization and analysis.

### Flow Cytometric Analysis

Hearts were flushed with PBS and the LV was then excised, minced, and digested with collagenase and DNase at 37 °C for 30 minutes.^20^ Flow cytometry was performed on a FACSCanto II cytometer (BD Bioscience) and data were analyzed using FlowJo software (BD). Macrophages were identified as lymphocyte common antigen-positive (CD45+), lymphocyte antigen 6 complex locus G6D-negative (Ly6G-), lymphocyte antigen-6C^low^-positive (Ly6C^lo^), and F4/80 antigen-positive (F4/80+) and further distinguished by cluster of differentiation 64 (CD64), major histocompatibility complex class II (MHCII), lymphatic vessel endothelial hyaluronan receptor 1 (Lyve1), and T cell immunoglobulin and mucin domain conataining 4 (TimD4) expression.^20^ Specifically, MHCII^lo^Lyvel+TimD4+ cells are cardiac resident macrophages.^21^^,^^22^

### Depletion of Macrophages In Vivo With Clodro

We hypothesize that macrophages, either derived from the greater omentum or alternatively from the circulation, are triggered by COP and account for the cardioprotective effects of COP. As a result, the reduction of macrophages should attenuate the beneficial results of COP. Clodro effectively depletes blood and tissue macrophages in mice in vivo.^23^ To examine whether macrophages are indispensable for the cardioprotective effect of COP, mice that received TAC and COP were intraperitoneally injected with Clodro at 5 mg/kg every 3 days for 8 weeks (n = 8 mice), starting on day 3 after COP surgery. Control mice were alternatively injected with 200 μL PBS-loaded liposomes (n = 8 mice).

### Statistical Analysis

The power analysis was used to estimate study sample size. Our pilot experiments showed that the value of mitral E/A ratio in C57BL/6 mice that underwent TAC is typically 1.20 ± 0.30, and mitral E/A ratio in C57BL/6 mice that underwent TAC and COP is approximately 1.55 ± 0.30. On the basis of an average standard deviation of 0.30, an n = 12 per group will allow for detection of a difference between groups at *P* < .05. Thus, 12 C57BL/6 mice per group were needed for the echocardiographic evaluation of cardiac function in mice.

For the continuous data, test of the normality was performed for deciding the measures of central tendency and statistical methods for data analysis. When continuous data follows a normal distribution, we present these data in mean and SD. Kruskal–Wallis test followed by Dunn test was used for multiple group comparisons. When a data were not normally distributed, medians and interquartile range are presented. Nonparametric Mann–Whitney test was used to compare 2 groups. All statistical analyses were performed using GraphPad Prism 8.

## Results

### C57BL/6 Mice Developed LV Hypertrophy 6 Weeks After TAC

**Figure E3:**
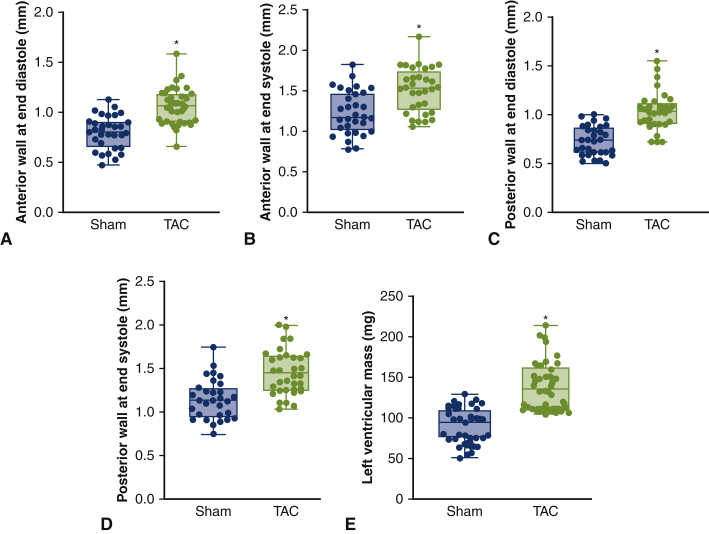
Left ventricular hypertrophy in C57BL/6 mice subjected to transverse aortic constriction (*TAC*) for 6 weeks. A, anterior wall at end diastole; (B) anterior wall at end systole; (C) posterior wall at end diastole; (D) posterior wall at end systole; and (E) left ventricular mass. Sham mice underwent all surgical procedures without constriction of the aorta. Parasternal short axis 2-chamber view-guided M-mode images of the left ventricle were used to quantitate left ventricular wall thickness and left ventricular mass. The *upper and lower borders of the box* represent the upper and lower quartiles. The *middle horizontal line* represents the median. The *upper and lower whiskers* represent the maximum and minimum values of nonoutliers. *Larger extra dots* represent outliers. *P* values were determined using 2-way repeated measures analysis of variance followed by post hoc analysis using Mann–Whitney test for comparison between 2 groups. ∗*P* < .05 versus sham (n = 31-32 mice per group).

**Figure E4:**
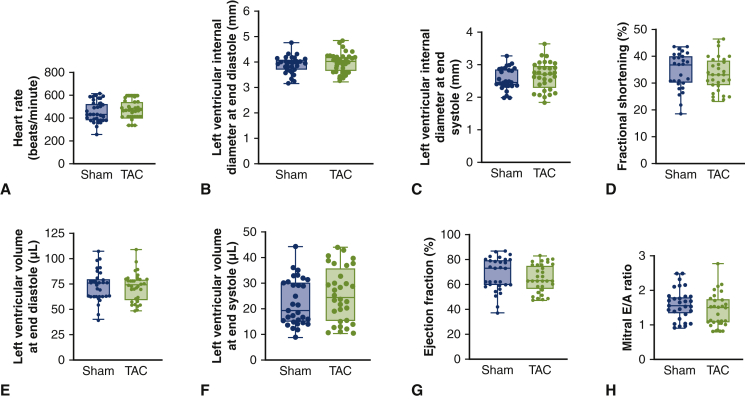
Echocardiographic parameters of mouse hearts 6 weeks after transverse aortic constriction (*TAC*). A, Heart rate; (B) left ventricular internal diameter at end diastole; (C) left ventricular internal diameter at end systole; (D) fractional shortening; (E) left ventricular volume at end diastole; (F) left ventricular volume at end systole; (G) ejection fraction; (H) mitral E/A ratio. The *upper and lower borders of the box* represent the upper and lower quartiles. The *middle horizontal line* represents the median. The *upper and lower whiskers* represent the maximum and minimum values of nonoutliers. *Extra dots* represent outliers. *P* values were determined using 2-way repeated measures analysis of variance followed by post hoc analysis using Mann–Whitney test for comparison between 2 groups. There were no differences in all echocardiographic parameters between TAC and sham groups (n = 31-32 mice per group).

Echocardiographic parameters of mice that underwent TAC for 6 weeks are shown in Figures E3 and E4. Compared with the control group, the thickness of the anterior wall at end diastole and end systole, posterior wall at end diastole and end systole, and LV mass were increased in the TAC group (*P* < .05; n = 31-32 mice per group; Figure E3). There were no differences in heart rate, LV internal diameter and volume, fractional shortening, ejection fraction, and mitral E/A ratio between sham control and TAC groups (Figure E4). This 6-week time point was before progression of diastolic dysfunction, and therefore served as our therapeutic window of opportunity.

### COP Ameliorated TAC-Induced Cardiac Hypertrophy and Dysfunction in Mice

**Figure 2:**
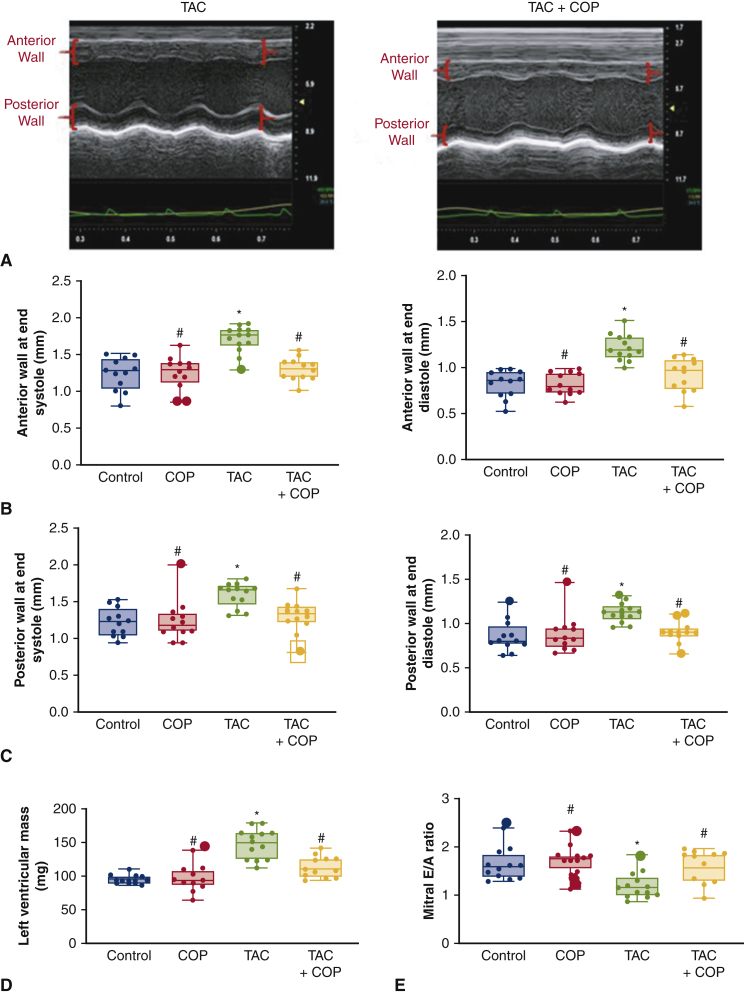
Cardio-omentopexy (*COP*) reduced transverse aortic constriction (*TAC*)-induced left ventricular hypertrophy and diastolic dysfunction. A, Representative echocardiographic M-mode images of the left ventricle; (B) anterior wall thickness at end systole and anterior wall thickness at end diastole; (C) posterior wall thickness at end systole and posterior wall thickness at end diastole; (D) left ventricular mass; and (E) mitral E/A ratio. Control mice were subjected to sham TAC surgery for 6 weeks and subsequent sham COP surgery for 8 weeks. COP mice were subjected to sham TAC surgery for 6 weeks and subsequent COP for 8 weeks. TAC mice underwent TAC for 6 weeks and subsequent sham COP surgery for 8 weeks. TAC+COP mice were subjected to TAC for 6 weeks and subsequent COP for 8 weeks. The *upper and lower borders* of the box represent the upper and lower quartiles. The *middle horizontal line* represents the median. The *upper and lower whiskers* represent the maximum and minimum values of nonoutliers. *Larger extra dots* represent outliers. *P* values were determined using 2-way repeated measures analysis of variance followed by post hoc analysis using Mann–Whitney test for comparison between 2 groups. ∗*P* < .05 versus control; #*P* < .05 versus TAC (n = 12-13 mice per group).

**Figure E5:**
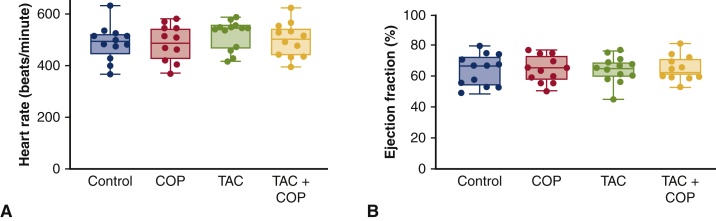
Heart rate (A) and systolic function (B) of mouse hearts 8 weeks after cardio-omentopexy (*COP*). Control mice were subjected to sham transverse aortic constriction (*TAC*) surgery for 6 weeks and subsequent sham COP surgery for 8 weeks. COP mice were subjected to sham TAC surgery for 6 weeks and subsequent COP for 8 weeks. TAC mice underwent TAC for 6 weeks and subsequent sham COP surgery for 8 weeks. TAC+COP mice were subjected to TAC for 6 weeks and subsequent COP for 8 weeks. The *upper and lower borders of the box* represent the upper and lower quartiles. The *middle horizontal line* represents the median. The *upper and lower whiskers* represent the maximum and minimum values of nonoutliers. *Extra dots* represent outliers. *P* values were determined using 2-way repeated measures analysis of variance followed by post hoc analysis using Mann–Whitney test for comparison between 2 groups. There were no differences in heart rate and ejection fraction between groups (n = 12-13 mice per group).

To determine if the greater omentum could ameliorate the progression of pressure overload-induced cardiac hypertrophy and dysfunction, we performed COP 6 weeks after TAC. At 8 weeks post COP, we compared cardiac function using echocardiography of all 4 groups: control, COP, TAC, and TAC+COP. As depicted in Figure E5, there were no differences in heart rate and ejection fraction among the 4 groups at the end of the 14-week time point. However, TAC at the 14-week time point led to increases in left ventricular mass versus control (Figure 2 and Table E1). Also elevated by TAC were anterior wall at end diastole, anterior wall at end systole, posterior wall at end diastole, and posterior wall at end systole thickness (*P* < .05; n = 12-13 mice per group). In contrast, mitral E/A ratio was reduced in TAC versus control. COP alone did not alter LV wall thickness, LV mass, and mitral E/A ratio (*P* > .05 COP vs control groups; n = 12 mice per group). However, COP attenuated TAC-induced increases in LV wall thickness and mitral E/A ratio (*P* < .05 between TAC+COP and TAC groups; n = 12-13 mice per group). These results indicate that COP reduces pressure overload-induced cardiac hypertrophy and improved LV diastolic function.

**Figure 3:**
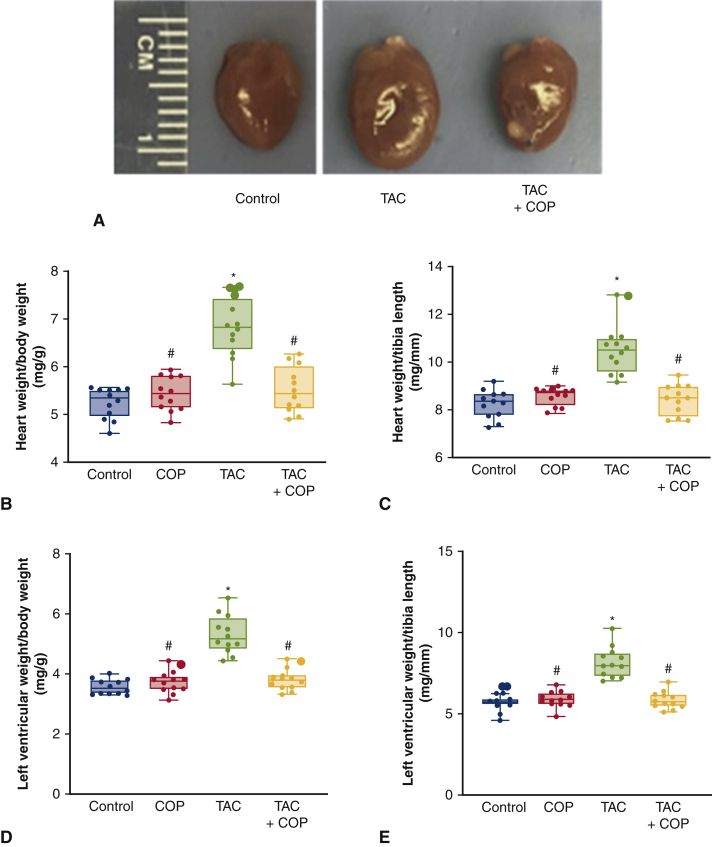
Cardio-omentopexy (*COP*) reduced transverse aortic constriction (*TAC*)-elicited increases in heart weight and left ventricular weight. A, gross pathology; (B) heart weight:body weight ratio; (C) heart weight:tibia length ratio; (D) left ventricular weight:body weight ratio; and (E) left ventricular weight:tibia length ratio. The mice of control, COP, TAC, and TAC+COP groups were treated as described in Figure 2. The *upper and lower borders* of the box represent the upper and lower quartiles. The *middle horizontal line* represents the median. The *upper and lower whiskers* represent the maximum and minimum values of nonoutliers. *Extra dots* represent outliers. *P* values were determined using 2-way repeated measures analysis of variance followed by post hoc analysis using Mann–Whitney test for comparison between 2 groups. ∗*P* < .05 versus control; #*P* < .05 versus TAC (n = 12 mice per group).

**Figure E6:**
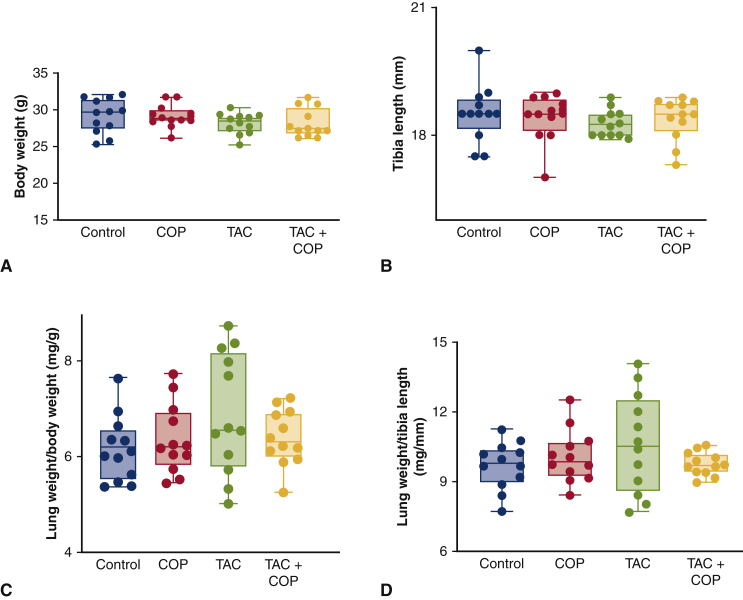
Physical weights and bone length of experimental groups at termination. A: body weight; B: tibia length; C: the ratio of lung weight/body weight: D: the ration of lung wright/tibia length. Control mice were subjected to sham transverse aortic constriction (*TAC*) surgery for 6 weeks and subsequent sham TAC surgery for 8 weeks. COP mice were subjected to sham TAC surgery for 6 weeks and subsequent cardio-omentopexy (*COP*) for 8 weeks. TAC mice underwent TAC for 6 weeks and subsequent sham COP surgery for 8 weeks. TAC+COP mice were subjected to TAC for 6 weeks and subsequent COP for 8 weeks. Lung tissue weight was normalized to body weight or tibia length. The *upper and lower borders of the box* represent the upper and lower quartiles. The *middle horizontal line* represents the median. The *upper and lower whiskers* represent the maximum and minimum values of nonoutliers. *Extra dots* represent outliers. *P* values were determined using 2-way repeated measures analysis of variance followed by post hoc analysis using Mann–Whitney test for comparison between 2 groups. There were no differences in body weight, tibia length, the ratios of both lung weight/body weight and lung weight/tibia length between groups (n = 12-13 mice per group).

We next harvested hearts and measured physical indices of hypertrophy. Gross examination of murine hearts revealed reductions in heart size post COP (Figure 3). Body weight and tibia length of mice were comparable among the 4 groups 8 weeks after COP (*P* > .05; n = 12 mice per group; Figure E6). We also compared the ratios of heart weight/body weight, heart weight/tibia length, LV weight/body weight, and LV weight/tibia length, which were increased in TAC only (Figure 3). Interestingly, these parameters were lower in the TAC+COP group relative to the TAC alone group (*P* < .05; n = 12 mice per group). There were no differences in the ratio of wet lung weight/body weight and lung weight/tibia length among the 4 groups.

### COP Attenuated TAC-Induced Increases in Cardiomyocyte Size and Myocardial Fibrosis

**Figure 4:**
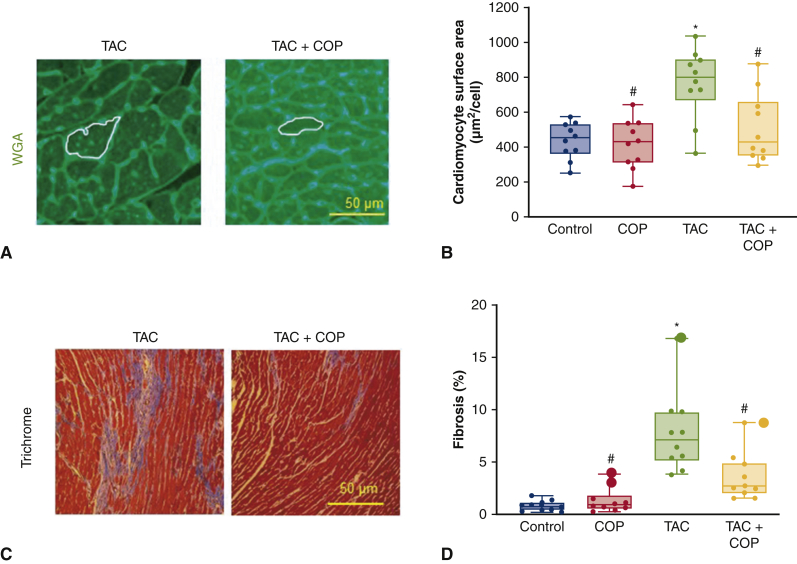
Cardio-omentopexy (*COP*) decreased the transverse artic constriction (*TAC*)-induced increases in cardiomyocyte size and myocardial fibrosis in C57BL/6 mice. A, Representative heart sections of TAC and TAC+COP mice stained with wheat germ agglutinin (WGA); B, quantification of cardiomyocyte surface area; C, representative heart sections stained with Masson's trichrome; and D, quantification of myocardial fibrosis. The mice of control, COP, TAC, and TAC+COP groups were treated as described in Figure 2. Scale bar = 50 μm. The *upper and lower borders* of the box represent the upper and lower quartiles. The *middle horizontal line* represents the median. The *upper and lower whiskers* represent the maximum and minimum values of nonoutliers. *Larger extra dots* represent outliers. *P* values were determined using 2-way repeated measures analysis of variance followed by post hoc analysis using Mann–Whitney test for comparison between 2 groups. ∗*P* < .05 versus control; #*P* < .05 versus TAC (n = 8-10 sections per group).

Mouse hearts were stained with WGA to quantitate cardiomyocyte surface area (Figure 4). Consistent with reduced heart size, cardiomyocyte size was also reduced (Figure 4). Cardiomyocyte surface area was greater in the TAC group relative to control groups (*P* < .05; n = 10 sections per group). COP did not alter cardiomyocyte surface area after sham TAC surgery (*P* > .05 between COP and control groups; n = 10 sections per group). However, COP decreased the TAC-induced increases in cardiomyocyte surface area (*P* < .05; n = 10 sections per group).

Myocardial interstitial fibrosis is an important contributor to left ventricular dysfunction in pressure overload-induced cardiac hypertrophy. Thus, we quantified myocardial interstitial fibrosis using Masson's trichrome stain. Sections obtained from control groups (sham TAC and COP alone) revealed little myocardial interstitial fibrosis (Figure 4). In contrast, sections obtained from TAC mice showed increased interstitial fibrosis (*P* < .05 between TAC and sham TAC groups; n = 10 sections per group). Compared with TAC sections, interstitial fibrosis was decreased in TAC+COP sections (*P* < .05; n = 10 sections per group).

### COP Promoted Angiogenesis in TAC

**Figure 5:**
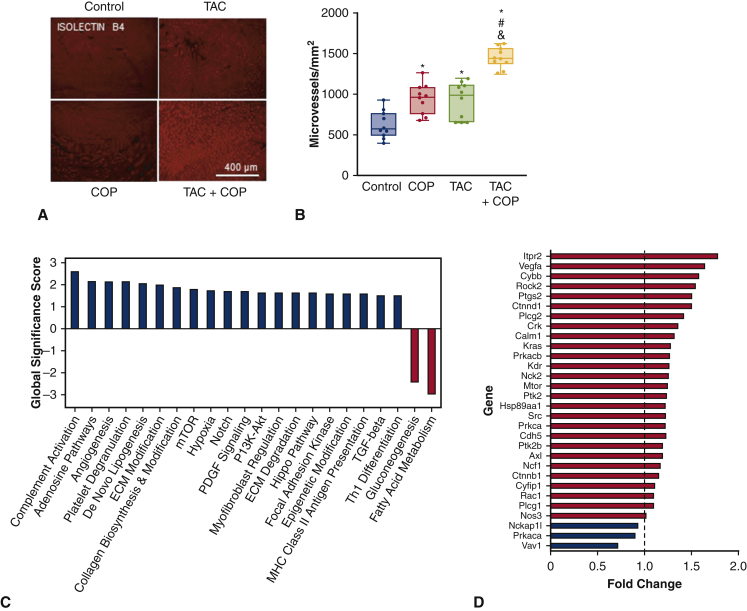
The effect of transverse aortic constriction (*TAC*) and cardio-omentopexy (*COP*) on microvessel density and gene expression profiling of mouse hearts. A, representative heart sections stained with isolectin B4 showing microvessels; B, quantification of microvessel density; C, global significance scores of pathways identified in fibrosis panel analysis. Only the most affected pathways, or pathways with a score >1.5 or less than −1.5, are shown; D, fold change of genes associated with angiogenesis. The mice of control, COP, TAC, and TAC+COP groups were treated as described in Figure 2. Scale bar = 400 μm. The *upper and lower borders* of the box represent the upper and lower quartiles. The *middle horizontal line* represents the median. The *upper and lower whiskers* represent the maximum and minimum values of nonoutliers. *P* values were determined using 2-way repeated measures analysis of variance followed by post hoc analysis using Mann–Whitney test for comparison between 2 groups. Values in panels (C) and (D) are for the TAC+COP group (n = 3) relative to the TAC group (n = 3). *ECM*, Extracellular matrix; *mTOR*, mammalian target of rapamycin; *PDGF*, platelet-derived growth factor; *PI3K-Akt*, phosphoinositide-3-kinase-protein kinase B; *Hippo*, hippocampal; *MHC*, major histocompatibility complex; *TGF*, transforming growth factor. ∗*P* < .05 versus control; #*P* < .05 versus TAC (n = 8 sections per group).

**Figure E7:**
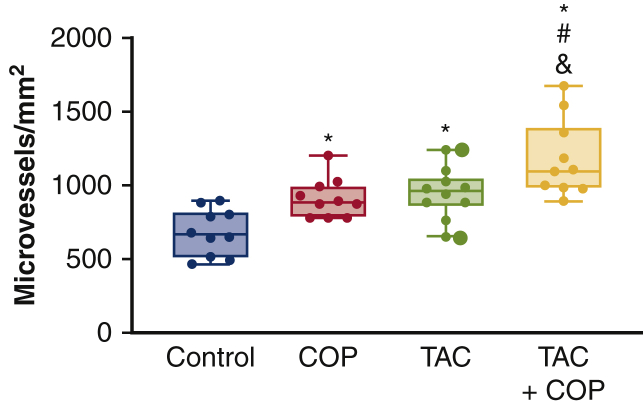
Quantification of microvessel density in mouse hearts stained with CD31 antibodies. Control mice were subjected to sham transverse aortic constriction (*TAC*) surgery for 6 weeks and subsequent sham cardio-omentopexy (*COP*) surgery for 8 weeks. COP mice were subjected to sham TAC surgery for 6 weeks and subsequent COP for 8 weeks. TAC mice underwent TAC for 6 weeks and subsequent sham COP surgery for 8 weeks. TAC+COP mice were subjected to TAC for 6 weeks and subsequent COP for 8 weeks. The *upper and lower borders of the box* represent the upper and lower quartiles. The *middle horizontal line* represents the median. The *upper and lower whiskers* represent the maximum and minimum values of nonoutliers. *Larger extra dots* represent outliers. *P* values were determined using 2-way repeated measures analysis of variance followed by post hoc analysis using Mann–Whitney test for comparison between 2 groups. ∗*P* < .05 versus control; #*P* < .05 versus COP; &*P* < .05 versus TAC (n = 10 sections per group).

The omentum has been shown in previous studies to induce angiogenesis. We therefore measured markers of microvessel density. Myocardial microvessels were increased to 946 ± 52 vessels per square millimeter in the TAC group and 913 ± 54 vessels per square millimeter in the COP alone group, from 626 ± 54 vessels per square millimeter in the control group, respectively (*P* < .05; n = 10 sections per group; Figure 5). Interestingly, the microvessel density was 1181 ± 82 vessels per square millimeter in the TAC+COP group (*P* < .05; TAC+COP vs control groups). CD31 antibody was used to stain vascular endothelial cells as an indicator of angiogenesis. As shown in Figure E7, the changes in coronary microvascular number were consistent with the results obtained from iB4-labeled microvessels.

To assess the bulk cardiac effects of COP, we performed unbiased gene array analysis of >700 genes known to be differentially regulated during tissue repair and fibrosis. Gene expression profile analysis confirmed induction of angiogenesis gene networks, among other networks, including increased expression of vascular endothelial growth factor A (VEGF-A) (Figure 5).

### COP Changed Myocardial Macrophages in TAC

**Figure 6:**
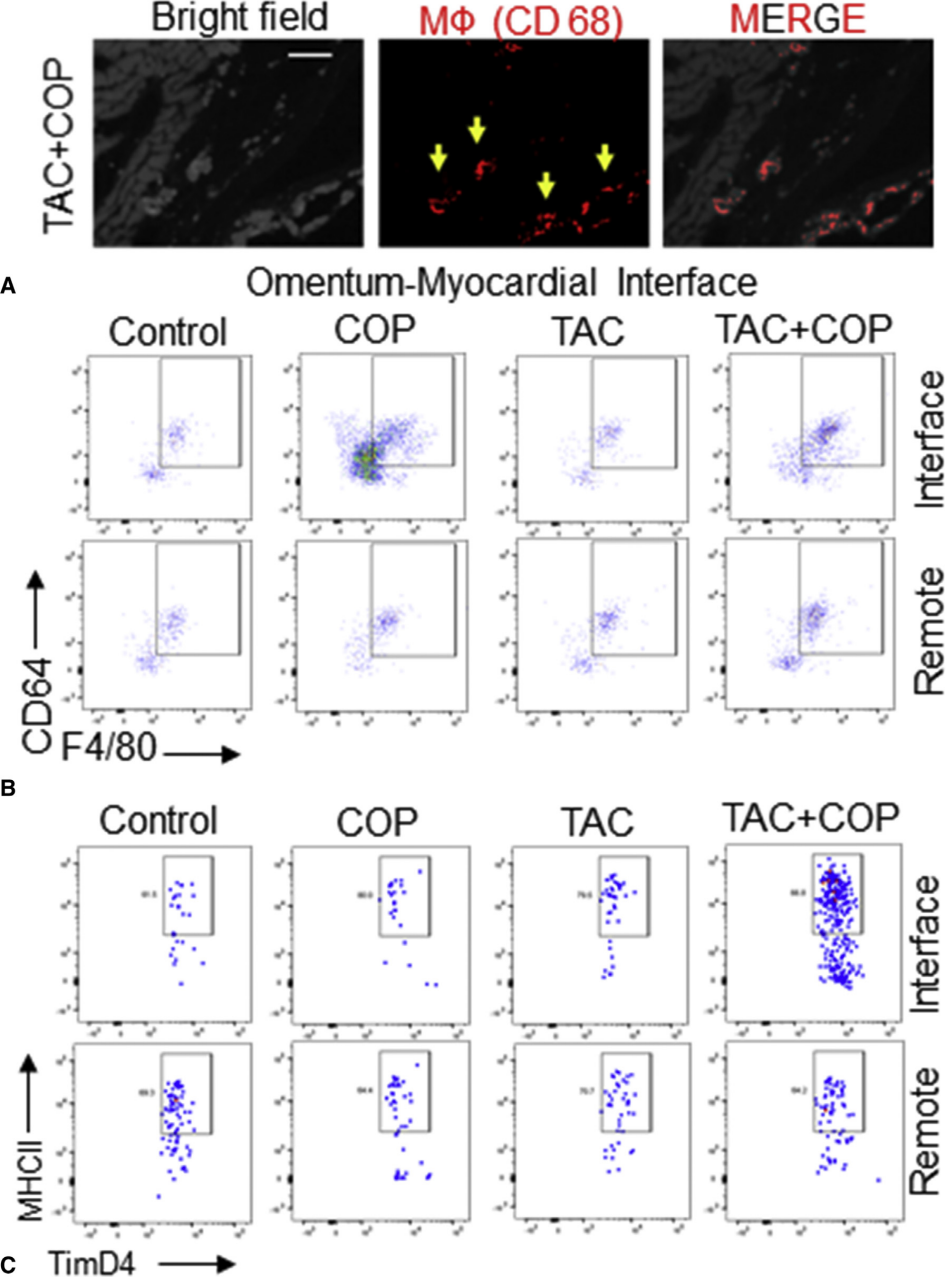
Cardio-omentopexy (*COP*) induces the accumulation of cardiac macrophages (*MФs*) at the myocardial-omentum junction. A, Representative images of MФ CD68 staining at the omental-myocardial junction (interface) in a mouse heart subjected to transverse aortic constriction (*TAC*) followed by COP; (B) flow cytometric analysis of total MФs (CD64-positive, F4/80-positive) in the mouse hearts of interface and remote regions; and (C) flow cytometric analysis of resident TimD4-positive MФs (MHCII-positive, TimD4-positive) in the mouse hearts of interface and remote regions. The mice of control, COP, TAC, and TAC+COP groups were treated as described in Figure 2.

**Figure E8:**
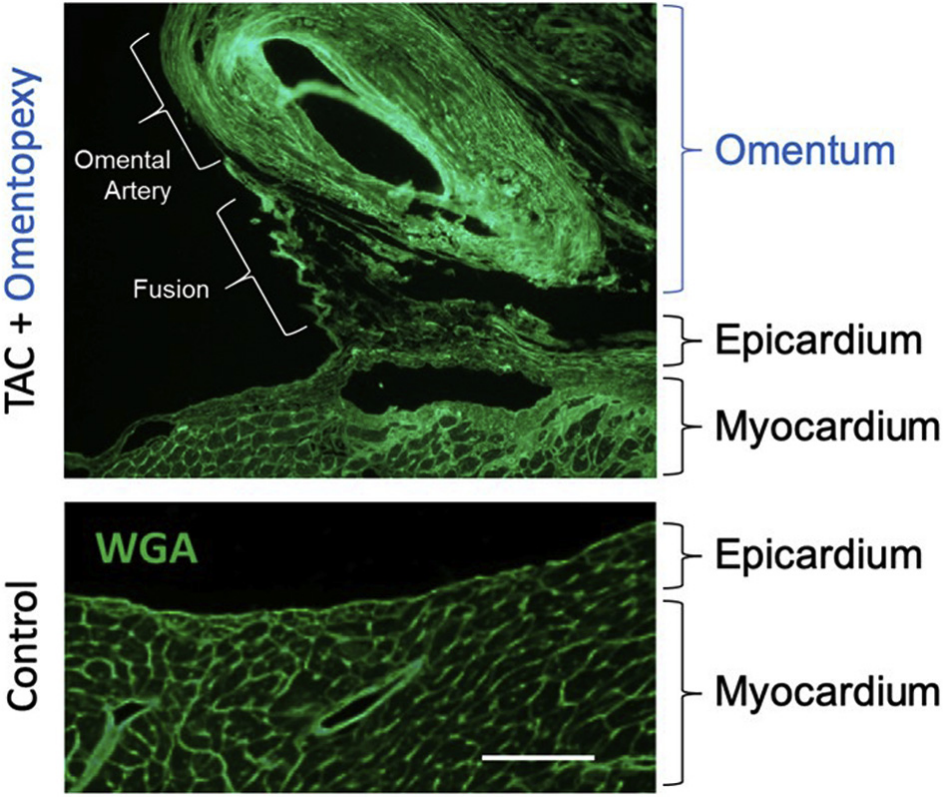
Evidence for omental fusion to myocardium in mouse. Displayed are histologic sections of control versus transaortic constriction (*TAC*) murine hearts followed by cardio-omentopexy. Tissue sections were stained with wheat germ agglutinin (*WGA*; *green*), which stains glycoproteins on cell membranes and matrix, to visualize cross cellular sectional area and connective tissue.

The omental adipose contains immune aggregates known as milky spots, which contain a constellation of immune cells and are conducive to the proliferation of mononuclear cells. Furthermore, inflammation is known to attract the migration of peritoneal macrophages. The function of omental macrophages might vary, and we considered the possibility that COP might mobilize reparative macrophages.^24^ Consistent with this premise, Figure 6 shows representative images of CD68+ macrophage accumulation at the omental–myocardial interface (Figure E8) in COP+TAC mice. To quantify macrophage accumulation, we performed flow cytometric analysis of the omentum–myocardial interface and remote myocardial extracts. Figure 6 illustrates the accumulation of CD64+F4/80+ macrophages in the omentum–myocardial interface. Moreover, flow cytometric analysis revealed clear elevations of cardiac MHCII^lo^Lyve1+TimD4+ resident macrophages that were specific to COP and localized to the omental–myocardial interface (Figure 6).

### Depletion of Macrophages With Clodro Blocked the Beneficial Effects of COP

**Figure 7:**
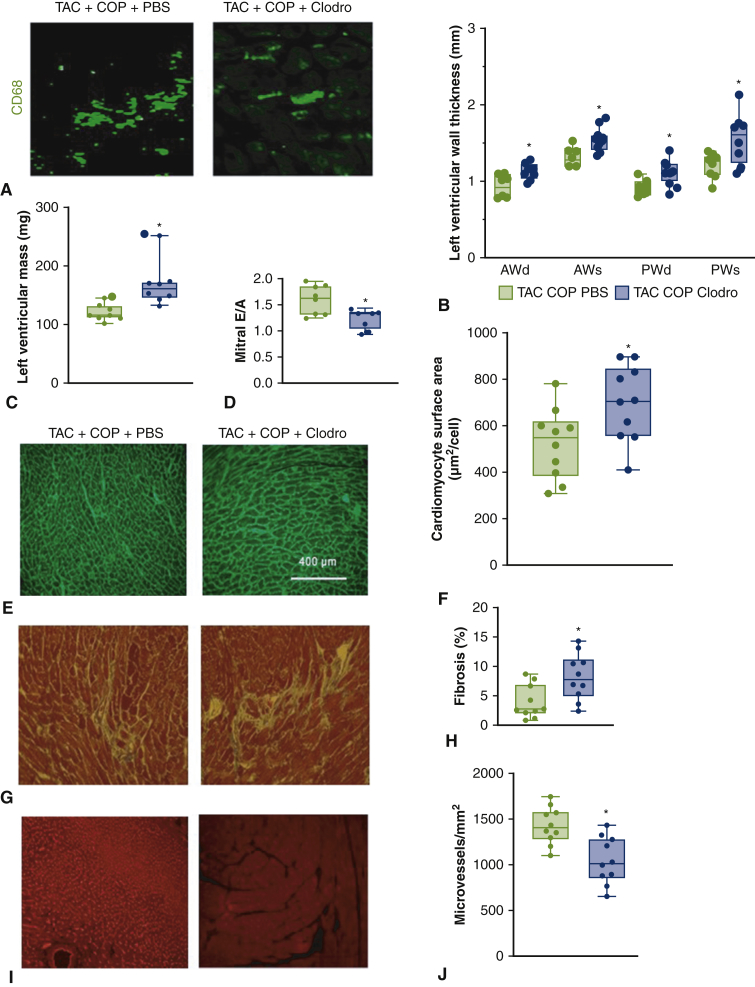
Depletion of macrophages with clodronate-liposomes (*Clodro*) impaired the cardioprotective effect of cardio-omentopexy (*COP*) in the mice subjected to transverse aortic constriction (*TAC*). A, CD68-stained heart sections showing macrophages in mouse hearts; and (B) quantification of left ventricular wall thickness; (C) left ventricular mass; (D) mitral E/A ratio; (E) wheat germ agglutinin-stained heart sections; (F) quantification of cardiomyocyte size; (G) Masson's trichrome-stained heart sections; (H) quantification of myocardial fibrosis; (I) isolectin B4-stained heart sections; and (J) quantification of microvessel density in mouse hearts. The mice in the TAC+COP+phosphate-buffered saline (PBS) group were subjected to TAC for 6 weeks and subsequent COP for 8 weeks and given PBS during 8 weeks of COP. The animals in the TAC+COP+Clodro group were subjected to TAC for 6 weeks and subsequent COP for 8 weeks and injected with Clodro during 8 weeks of COP. Scale bar = 400 μm. The *upper and lower borders* of the box represent the upper and lower quartiles. The *middle horizontal line* represents the median. The *upper and lower whiskers* represent the maximum and minimum values of nonoutliers. *Larger extra dots* represent outliers. *P* values were determined using 2-way repeated measures analysis of variance followed by post hoc analysis using Mann–Whitney test for comparison between 2 groups. *PBS*, Phosphate-buffered saline; *AWd*, anterior wall at end diastole; *AWs*, anterior wall at end systole; *PWd*, posterior wall at end diastole; *PWs*, posterior wall at end systole. ∗*P* < .05 versus TAC+COP+PBS (n = 8-10 per group).

We next asked if macrophages, either derived from the greater omentum or alternatively from the circulation, are triggered by COP and required for cardioprotection. To test this hypothesis, it was important for us to temporally deplete macrophages post COP, because macrophages have also been shown to regulate TAC-associated cardiac dysfunction per se.^25^ In this context, previous reports have documented omental and circulatory macrophage depletion after intraperitoneal injection of Clodro.^26^ We therefore injected Clodro post COP and subsequently monitored the morphology and function of the left ventricle. Clodro was effective in depleting cardiac macrophages (Figure 7). Furthermore, protection from LV hypertrophy and cardiac diastolic dysfunction were lost after Clodro treatment (Figure 7 and Table E2). Also lost were the COP-induced decreases in cardiomyocyte size and myocardial fibrosis, and COP-induced elevations in microvessels. These results suggest that macrophages are essential for the cardioprotective effect of COP.

## Discussion

**Figure E9:**
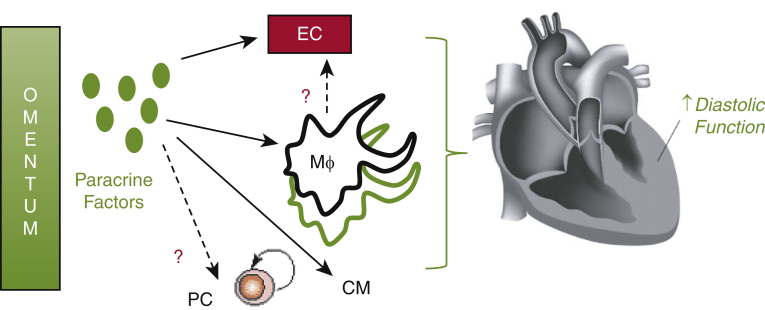
Candidate pathways by which the omentum might protect the heart after cardio-omentopexy. The omentum might secrete paracrine factors to mobilize cardioprotective endothelial cells (*ECs*) during angiogenesis. Alternatively, such factors might trigger reparative macrophages (*Mɸs*) in the heart. It is unclear if such macrophages originate from the omentum, the circulation, or are resident in the heart. Another scenario is that the omentum might mobilize protective progenitor cells (*PCs*). *CM*, Cardiomyocyte.

Our findings describe a new murine model to understand the benefits of COP (Video 1). Through this model, we propose a new mechanism for COP-mediated cardioprotection (Figure E9). This includes the activation of a proreparative inflammatory response accompanied by cardiac angiogenesis and reductions in cardiac hypertrophy and cardiomyocyte cell size (Video Abstract). Our data are also consistent with the premise that macrophages are central protagonists in COP-induced cardioprotection.

**Figure 8:**
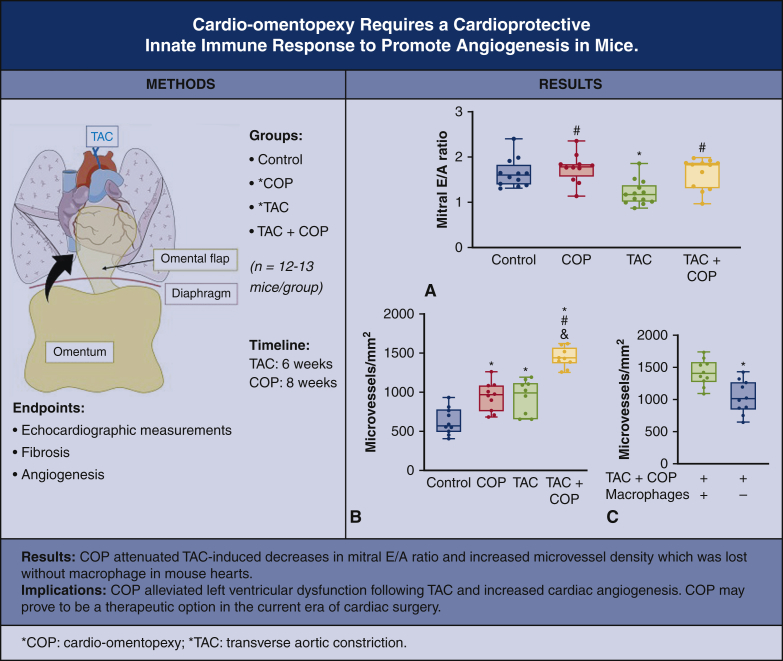
Key study methods, results, and implications. A, Left ventricular diastolic function (mitral E/A ratio) measured from echocardiography; B, changes in myocardial angiogenesis (microvessel density measured from the biotinylated-isolectin B4-stained mouse hearts); C, effects of macrophage depletion with clodronate-liposome on myocardial angiogenesis in the mice subjected to transverse aortic constriction (*TAC*) and cardio-omentopexy (*COP*). Control mice were subjected to sham TAC surgery for 6 weeks and subsequent sham COP surgery for 8 weeks. COP mice were subjected to sham TAC surgery for 6 weeks and subsequent COP for 8 weeks. TAC mice underwent TAC for 6 weeks and subsequent sham COP surgery for 8 weeks. TAC+COP mice were subjected to TAC for 6 weeks and subsequent COP for 8 weeks. The *lower and upper borders* of the box represent the lower and upper quartiles (25th percentile and 75th percentile). The *middle horizontal line* represents the median. The *lower and upper whiskers* represent the minimum and maximum values of nonoutliers. Larger *extra dots* represent outliers.

It has been proposed that angiogenesis in the early phase of pressure overload allows for cardiac hypertrophy and the maintenance of cardiac function, whereas the maladaptive phase is characterized by a regression in microvessel density, hypertrophy, and systolic function.^27^ Consistent with early phase findings, we observed cardiac hypertrophy and increased angiogenesis in TAC mice. Interestingly, COP prevented diastolic dysfunction without the induction of cardiac hypertrophy. It is possible that the increased angiogenesis seen in COP is responsible for cardioprotection via increased nutrient delivery, delaying or preventing cardiac dysfunction (Figure 8). Elucidating the mechanism of COP cardioprotection, including how COP affects cardiomyocytes, is of importance for future studies.

In non-COP mouse hearts, MHCII^lo^Lyve1+TimD4+ cells are a cardiac resident macrophage population.^21^^,^^28^^,^^29^ Cardiac resident macrophages participate in angiogenesis, because these cells can produce VEGF-A during tissue repair.^30^ Additionally, evidence suggests that MHCII^lo^Lyve1+TimD4+ macrophages play important roles in angiogenesis and efferocytosis.^21^^,^^22^ We observed that COP enhanced MHCII^lo^Lyve1+TimD4+ macrophages localized to the omental–myocardial interface, therefore it is reasonably believed that COP induces an increase in MHCII^lo^Lyve1+TimD4+ macrophages, which contribute to the cardioprotection.

Macrophages are known residents of the greater omentum. Recently, a new subtype of omental macrophage has been described,^31^ defined as a transcriptionally distinct CD163+TimD4+ population of resident omental macrophages. These CD163+TimD4+ macrophages are long-lived, of embryonic origin, and include gene expression profiles specific for angiogenesis, regeneration, and endocytosis.^32^ It remains unclear whether omental CD163+TimD4+ macrophages are associated with COP-induced increases in cardiac MHCII^lo^Lyve1+TimD4+ macrophages. It will be of interest to determine if these CD163+TimD4+ macrophages are directly responsible for cardioprotection, or if the greater omentum secretes paracrine factors to mobilize circulating macrophages.

### Limitations

As in most studies, our experimental approach includes limitations. This includes the use of liposomal Clodro, which might have off-target effects. Therefore, future corroborative strategies, such as genetic-based approaches, might be applied. Although these approaches will add causal evidence for the role of myeloid cells during COP, our current opinion is that the more clinically relevant and interesting future direction is to determine the sufficiency, source, and origin of protective omental factors. Separately, further useful information will come from incorporating additional clinically relevant metrics, such as complementary longitudinal measurements with magnetic resonance imaging, molecular positron emission tomography, and LV pressure after COP. Nevertheless, it is important to stress that the new murine cardioprotective COP phenotype is consistent with our previously reported COP-mediated cardioprotection in rats.^10^ This indicates a reproducible consistency in rodents and that the therapeutic potential of COP is conserved between species. The generation of the murine COP model is the first step in leveraging the array of experimental tools in the mouse to understand COP cardioprotection at the molecular level. This insight might then be leveraged for use in larger animals and ultimately in patients with hypertrophic cardiomyopathy (HCM) or diastolic heart failure.

## Conclusions

Taken together, further studies are warranted to optimize therapeutic strategies of COP. This includes testing COP in larger animals as a step toward clinical application. HCM is a disease in which the heart muscle becomes abnormally hypertrophied. The thickened heart muscle can make it harder for the heart to pump blood. Several different surgeries or procedures are currently available to treat HCM or its symptoms. They range from open heart surgery to implantation of a device to control heart rhythm. Septal myectomy helps improve blood flow out of the heart and reduces mitral regurgitation. The present study suggests that the combination of septal myectomy and COP might provide better outcomes than septal myectomy alone. Furthermore, complementary strategies, such as preactivating the greater omentum, might improve therapeutic potential.^2^^,^^33^ Alternatively, the isolation of cardioprotective cells and factors from the greater omentum might be an approach for cellular therapy or synergy with cardiac tissue bioscaffolds.^3^^,^^34^^,^^35^ Last, the identification of additional protective cellular populations from the greater omentum might be achieved by single-cell mRNA sequencing.

### Conflict of Interest Statement

The authors reported no conflicts of interest.

The *Journal* policy requires editors and reviewers to disclose conflicts of interest and to decline handling or reviewing manuscripts for which they may have a conflict of interest. The editors and reviewers of this article have no conflicts of interest.

## Figures and Tables

**Table E1:** Echocardiographic parameters of C57BL/6 mice

	Control (n = 12)	COP (n = 12)	TAC (n = 13)	TAC with COP (n = 12)
Left ventricle				
Heart rate, beats per minute	490 ± 66	487 ± 67	522 ± 55	501 ± 63
Anterior wall at end diastole, mm	0.82 ± 0.14	0.83 ± 0.12	1.22 ± 0.14∗†	1.00 ± 0.17∗‡
Anterior wall at end systole, mm	1.24 ± 0.21	1.25 ± 0.22	1.71 ± 0.18∗†	1.31 ± 0.15∗‡
Posterior wall at end diastole, mm	0.86 ± 0.17	0.89 ± 0.20	1.12 ± 0.10∗†	0.90 ± 0.11∗‡
Posterior wall at end systole, mm	1.22 ± 0.18	1.24 ± 0.27	1.59 ± 0.16∗†	1.30 ± 0.21∗‡
LV internal diameter at end diastole, mm	3.93 ± 0.28	3.76 ± 0.31	3.79 ± 0.31	3.97 ± 0.36
LV internal diameter at end systole, mm	2.64 ± 0.33	2.51 ± 0.24	2.58 ± 0.42	2.66 ± 0.33
Fractional shortening, %	33 ± 6	33 ± 4	32 ± 7	33 ± 4
LV end diastolic volume, μL	68 ± 15	62 ± 7	61 ± 11	69 ± 14
LV end systolic volume, μL	24 ± 9	21 ± 5	22 ± 8	25 ± 10
Ejection fraction, %	64 ± 10	65 ± 8	65 ± 8	65 ± 8
LV mass, mg	96 ± 6	96 ± 18	148 ± 21∗†	114 ± 15∗‡
Cardiac output, mL/min	19 ± 5	18 ± 3	20 ± 4	21 ± 3
Mitral E/A ratio	1.64 ± 0.31	1.75 ± 0.29	1.21 ± 0.26∗†	1.63 ± 0.32
Aorta				
Peak velocity of aortic valve, m/s	1.27 ± 0.26	1.24 ± 0.20	1.15 ± 0.23	1.19 ± 0.26
Transverse aortic lumen diameter, mm	1.39 ± 0.32	1.39 ± 0.24	1.77 ± 0.32∗†	1.69 ± 0.25∗†
TAC pressure gradient, mm Hg	2.8 ± 0.8	2.7 ± 0.5	59.5 ± 30.7∗†	57.1 ± 28.6∗†

Data are presented as mean ± SD. Control mice were subjected to sham TAC surgery for 6 weeks and subsequent sham COP surgery for 8 weeks. COP mice were subjected to sham TAC surgery for 6 weeks and subsequent COP for 8 weeks. TAC mice underwent TAC for 6 weeks and subsequent sham COP surgery for 8 weeks. TAC with COP mice were subjected to TAC for 6 weeks and subsequent COP for 8 weeks. Kruskal–Wallis test followed by Dunn's test was used to analyze multiple group comparisons. *COP*, Cardio-omentopexy; *TAC*, transverse aortic constriction; *LV*, left ventricular.

∗ Indicates *P* < .05 versus control (n = 12-13 mice per group).

† Indicates *P* < .05 versus COP (n = 12-13 mice per group).

‡ Indicates *P* < .05 versus TAC (n = 12-13 mice per group).

**Table E2:** Echocardiographic and physical parameters of C57BL/6 mice that received clodronate-liposome treatment

	TAC with COP and PBS (n = 8)	TAC with COP and Clodro (n = 8)
Heart rate, beats per minute	483 ± 61	496 ± 84
Anterior wall at end diastole, mm	0.97 ± 0.15	1.13 ± 0.11∗
Anterior wall at end systole, mm	1.34 ± 0.11	1.52 ± 0.14
Posterior wall at end diastole, mm	0.91 ± 0.10	1.12 ± 0.17∗
Posterior wall at end systole, mm	1.20 ± 0.16	1.56 ± 0.34∗
Left ventricular internal diameter at end diastole, mm	4.27 ± 0.31	4.38 ± 0.39
Left ventricular internal diameter at end systole, mm	2.90 ± 0.26	3.11 ± 0.47
Fractional shortening, %	32 ± 3	29 ± 7
Left ventricular end diastolic volume, μL	83 ± 13	91 ± 14
Left ventricular end systolic volume, μL	31 ± 8	38 ± 17
Ejection fraction, %	63 ± 6	59 ± 15
Left ventricular mass, mg	120 ± 14	168 ± 37∗
Cardiac output, mL/min	23 ± 3	26 ± 6
Mitral E/A ratio	1.59 ± 0.28	1.23 ± 0.19∗
Heart weight, mg	162 ± 12	208 ± 15∗
Heart weight/body weight, mg/g	5.9 ± 0.3	6.8 ± 0.3∗
Heart weight/tibia length, mg/mm	9.9 ± 0.63	11.5 ± 0.98∗
Left ventricle weight/body weight, mg/g	4.3 ± 0.4	5.0 ± 0.4∗

Data are presented as mean ± SD. The mice in the TAC with COP and PBS group were subjected to TAC for 6 weeks and subsequent COP for 8 weeks and given PBS during 8 weeks of COP. The animals in the TAC with COP and Clodro group were subjected to TAC for 6 weeks and subsequent COP for 8 weeks and injected with Clodro during 8 weeks of COP. *P* values were determined using 2-way repeated measures analysis of variance followed by post hoc analysis using the Kruskal–Wallis test for comparisons between 2 groups. *TAC*, Transverse aortic constriction; *COP*, cardio-omentopexy; *PBS*, phosphate-buffered saline; *Clodro*, clodronate-liposome.

∗ Indicates *P* < .05 versus TAC with COP and PBS (n = 8 mice per group).

## Supplementary Data

Online Data Supplement

Video Abstract Video available at: https://www.jtcvs.org/article/S2666-2736(22)00096-1/fulltext.

Video 1 Cardio-omentopexy requires a cardioprotective innate immune response to promote myocardial angiogenesis. The video describes the background, hypothesis, methods, results, and conclusions of this study. It will be presented in the American Heart Association Scientific Sessions 2021 in Boston, Massachusetts. Video available at: https://www.jtcvs.org/article/S2666-2736(22)00096-1/fulltext.
