# Supplementary material for: Cardio-omentopexy requires a cardioprotective innate immune response to promote myocardial angiogenesis in mice
Source: JTCVS Open. 2022 Feb 24;10:222–42. doi: 10.1016/j.xjon.2022.02.027 (PMC9390370; doi:10.1016/j.xjon.2022.02.027)
Supplement: Online Data Supplement [file mmc1.docx]

**SUPPLEMENTAL MATERIAL**

**Cardio-Omentopexy Requires a Cardioprotective Innate**

**Immune Response to Promote Myocardial Angiogenesis in Mice**

**Zhi-Dong Ge^1^, Riley M. Boyd^1^, Connor Lantz^1^, Edward B. Thorp^1^, and Joseph M. Forbess^2^**

^1^The Heart Center and Cardiovascular-Thoracic Surgery, Stanley Manne Children’s Research Institute, Ann & Robert H. Lurie Children’s Hospital of Chicago, Feinberg School of Medicine, Northwestern University, 255 East Chicago Road, Chicago, Illinois 60611, USA; and ^2^Department of Surgery, University of Maryland School of Medicine and The Children’s Heart Program, University of Maryland Children’s Hospital, Baltimore, Maryland 21201, USA.

**METHODS**

**Animals**

The general structure of the greater omentum in adult mice is similar to that in human fetus and in children.^11^ The use of mice in pre-clinical studies has proven effecive in the research of many cardiovascular diseases. We used C57BL/6 mice from the Jackson Laboratory (Bar Harbor, ME) in the current study. Male C57BL/6 mice **(20-25g; 8-10 weeks)** were used in bred and cared for in the Animal Facility of Northwestern University Chicago Campus. The animals were kept on a 12-h light-dark cycle in a temperature-controlled room. Animal care and all experimental procedures were performed in accordance with the NIH Guide for the Care and Use of Laboratory Animals (Institute for Laboratory Animal Research, National Academy of Sciences, USA, 8th edition, 2011), and experimental protocols were approved by the Institutional Animal Care and Use Committee at Northwestern University (Chicago, IL) (The protocol number: IS000010625, the date of approval: January 10, 2019).

**Experimental outline**

The experimental protocol is outlined in Figure 1. The effects of COP on pressure overload-induced hypertrophy and cardiac dysfunction were determined in C57BL/6 mice randomly assigned to 4 experimental groups: control (n = 12 mice), COP (n = 12 mice), transverse aortic constriction (TAC; n = 13 mice), and TAC+COP (n = 12 mice) (Figure 1A). Control mice were subjected to sham transverse aortic constriction (TAC) surgery for 6 weeks and subsequent sham COP surgery for 8 weeks (COP). The mice in the COP group were subjected to sham TAC surgery for 6 weeks and subsequent COP for 8 weeks. The animals in the TAC group underwent TAC for 6 weeks and subsequent sham COP surgery for 8 weeks. In the TAC + COP group, the mice were subjected to TAC for 6 weeks and subsequently COP for 8 mice. The morphology and function of the left ventricle (LV) at baseline (3 days before TAC or sham TAC surgery), 6 weeks post-TAC, and 8 weeks post-COP were evaluated with an echocardiography. Myocardial fibrosis, cardiomyocyte size, microvessel density, and cardiac macrophages were quantified at 8 weeks after COP or sham surgery. The role of cardiac macrophages in the cardioprotective effect of COP was examined in COP+TAC mice through depletion of macrophages with clodronate-liposomes (Clodro) (Figure 1B).

**TAC**

Minimally invasive TAC was performed on male C57BL/6 mice (n = 25, 13 mice in TAC and 12 mice in TAC + COP group) at 8-10 weeks of age, as described.^12^ Under the anesthesia of 1.5-2.0% isoflurane, a 0.5 cm horizontal incision was made at the level of the suprasternal notch. The chest was opened 2-3 cm in the proximal portion of the sternum. Through the optics of a surgical microscope (Zeiss, Stuttgart, Germany), the thymus was deflected to expose the aortic arch. The transverse aorta was constricted between the innominate and left common carotid arteries by a 7-0 prolene suture ligature tied against a 25-gauge blunted needle (Supplementary Figures 1 and 2). The latter was quickly removed to yield a constriction of 0.4 mm in diameter, which was measured with a VisualSonics Vevo 3100 High-resolution Imaging System (Toronto, Canada).^13^ Sham control mice (n = 24, 12 mice in control group and 12 mice in TAC group) underwent all surgical procedures without aortic constriction. The chest was closed, mice were removed from the ventilator, and kept in a warming chamber until recovery. Body temperature was maintained at 37.0-37.5 °C throughout the experiment.

**Murine COP**

Six weeks after TAC or sham TAC surgery, male C57BL/6 mice (n = 24, 12 mice in COP and 12 mice in TAC + COP group) were initially anesthetized with isoflurane (approximately 4%). Once consciousness was lost, the trachea was cannulated with a 25-gauge flexible catheter. The catheter was connected to a mechanical ventilator that provided positive-pressure ventilation. The ventilator was connected to a vaporizer (Braintree Scientific, Inc., Braintree, MA) delivering ~1.5% isoflurane. Mice were placed in the dorsal decubitus position on a warming plate to maintain a body temperature of approximately 37ºC. After shaving, the surgical areas were scrubbed and disinfected, as previously described.^14^ To prepare the pedicled omental flap, a transverse 1 cm skin incision was made in the left upper quadrant of the abdomen and laparotomy was performed.^15^ One flap with the right gastroepiploic artery was made and translocated into the chest through the diaphragm. A left thoracotomy was performed between the 4th and 5th ribs, and the lungs were retracted to expose the heart.^16^ The pedicled omental flap was sutured to the LV anterior wall with a 7-0 prolene suture (Supplementary Figure 2). The lungs were inflated by positive end-respiratory pressure. The chest was closed. Sham-operated animals (n = 25, 12 mice in control and 13 mice in TAC group) underwent the same procedure except for the suturing of the omental flap. Animals were kept in a warm chamber until recovery.

**Transthoracic echocardiography**

Non-invasive transthoracic echocardiography was used to evaluate left ventricular geometry and function in mice at baseline (3 days prior to TAC or sham TAC surgery), 6 weeks post-TAC, and 8 weeks post-COP using 1.5% isoflurane. Echocardiography was performed with a VisualSonics Vevo 3100 High-resolution Imaging System.^17^ M-mode images were recorded from the parasternal short axis 2-chamber view at the papillary muscle level. Heart rate, LV anterior wall and interventricular septum thickness, and LV internal diameter were measured. Fractional shortening was calculated using: (LV end-diastolic diameter–LV end-systolic diameter)/LV end-diastolic diameter×100. Pulsed Doppler waveforms recorded in the apical-4-chamber view were used for the measurement of the peak velocities of mitral E and A waves.

**Histopathological examination of mouse hearts**

To validate the findings from echocardiographic examination of mice, mice at 8 weeks after COP or sham COP surgery were euthanized, and mouse hearts were visualized and weighed. Dehydrated mouse hearts were embedded with paraffin and sliced transversely from the apex to the basal part of the LV at 4-5 μm-thickness.^13^ Sections were stained with Masson’s trichrome to assess myocardial fibrosis, wheat germ agglutinin (WGA) for cardiomyocyte surface area, biotinylated-isolectin B4 (iB4) and CD31 for quantification of myocardial microvessels, or CD68 for visualization of macrophages (n=10 sections/mouse, 3 mice/group). Stained sections were imaged with an Olympus microscope (Olympus America, Melville, New York, USA) at 200× magnification or as indicated. Image analysis was completed with ImageJ software.

*Masson’s Trichrome staining.* 2,2,2-tribromoethanol-anesthetized mice received intracardiac saturated KCl (30 mmol/L and 5% dextrose in 1× phosphate-buffered saline [PBS]) to arrest the heart in diastole, as described.^18^ Mouse hearts were washed with cold PBS, fixed with 10% Formalin, dehydrated, and embedded in paraffin. The percentage of total fibrosis area was calculated as the summed, blue-stained regions of interest divided by total area.

*Immunohistochemical staining*. Cardiomyocyte size and myocardial microvessel density were measured by staining mouse hearts with WGA and iB4, respectively.^19^ Mouse hearts were fixed with 4% paraformaldehyde then embedded in paraffin. Sections from the midpoint of the left ventricle were deparaffinized and hydrated. For measurements of cardiomyocyte size, tissue sections were stained with fluorescence Oregon Green 488–labeled WGA. For the determination of myocardial microvessel density, the slide was rehydrated and boiled to 95°C in 0.01 mol/L sodium citrate solution (pH 6.0). After cooling to room temperature, the slide was balanced with PBLEC solution (PBS + 0.01 mol/L CaCl2, MgCl2, MnCl2, pH 6.8) and stained with iB4 overnight. Microvascular density was counted and compared between.

*Immunofluorescent staining.* Immunofluorescent staining with CD31 and CD68 antibodies was used to detect vascular endothelial cells and macrophages. Hearts were embedded in optimum cutting temperature (OCT) freezing medium; 10-mm transverse sections were cut using a cryostat (Cryo Star NX70, Thermo Fisher Scientific, Waltham, MA, USA). Sections were fixed in 4% paraformaldehyde, permeabilized in saline containing 0.5% Tween-20, and blocked in 10% FBS at room temperature. Primary antibodies against CD31 and CD68 antigens were added and incubated at 37ºC. Appropriate secondary antibodies were then added and incubated at 37^o^C.

**Nanostring gene array**

Myocardium from TAC (n = 3) and TAC followed by COP (TAC+COP) (n=3) mice were dissected and stored in RNAlater (Thermo Fisher Scientific). Total RNA was extracted with TRIzol reagent (Invitrogen, Carlsbad, CA, USA) following the manufacturer’s protocol. Intact RNA (50 ng) was used for Nanostring analysis with the nCounter Fibrosis Panel (Seattle, Washington, USA). nSolver 4.0 (Nanostring Technologies, Seattle, WA, USA) was utilized for raw count normalization and analysis. Data visualization was completed with R Studio (v1.3) using the ggplot2, ComplexHeatmap, dendextend, and circilize packages.

**Flow cytometric analysis**

Hearts were flushed with PBS and the LV was then excised, minced, and digested with collagenase and DNase at 37°C for 30 minutes.^20^ Hearts were triturated through a 40-μm cell strainer. Red blood cells were lysed, and total viable cells were determined by Trypan blue staining. Cells were incubated with Fc Block (Biolegend, San Diego, CA, USA) for 15 minutes and labeled with fluorescently conjugated antibodies. Flow cytometry was performed on a FACSCanto II cytometer (BD Biosciences, San Jose, CA, US) and data were analyzed by FlowJo software (Tree Star, Inc., Ashland, OR, USA). Macrophages were identified as CD45+Ly6G-Ly6CloF4/80+ and further distinguished by CD64, MHCII, Lyve1, and TimD4 expression.^20^ Specifically, MHCII^lo^Lyvel+TimD4+ cells are cardiac resident macrophages.^21,22^

**Depletion of macrophages *in vivo with Clodro***

We hypothesize that macrophages, either derived from the greater omentum or alternatively from the circulation, are triggered by COP and account for the cardioprotective effects of COP. As a result, the reduction of macrophages should attenuate the beneficial results of COP. Clodro effectively depletes blood and tissue macrophages in mice *in vivo*.^23^ To examine whether macrophages are indispensable for the cardioprotective effect of COP, mice receiving both TAC and COP were intraperitoneally injected with Clodro (Haarlem, North Holland, the Netherlands) at 5 mg/kg every three days for 8 weeks (n = 8 mice), starting on days three after COP surgery. Control mice were alternatively injected with 200 μL PBS-loaded liposomes (n = 8 mice).

**Statistical Analysis**

The power analysis was used to estimate study sample size. Our pilot experiments showed that the value of mitral E/A ratio in C57BL/6 mice undergoing TAC is typically 1.20 ± 0.30, and mitral E/A ratio in C57BL/6 mice undergoing TAC+COP is around 1.55 ± 0.30. Based upon an average standard deviation of 0.30, an n = 12 per group will allow for detection of a difference between groups at P < 0.05.  Thus, 12 C57BL/6 mice per group were needed for the echocardiographic evaluation of cardiac function in mice.

For the continuous data, test of the normality was performed for deciding the measures of central tendency and statistical methods for data analysis. When a continuous data follows normal distribution, we present this data in mean and SD. Kruskal-Wallis test followed by Dunn’s test was used for multiple group comparisons. When a data was not normally distributed, medians and interquartile range were presented. Non-parametric Mann Whitney test was used to compare two groups. All statistical analyses were performed using GraphPad Prism 8 (GraphPad Software, Inc., San Diego, CA). A value of p less than 0.05 (two tailed) was considered statistically significant.


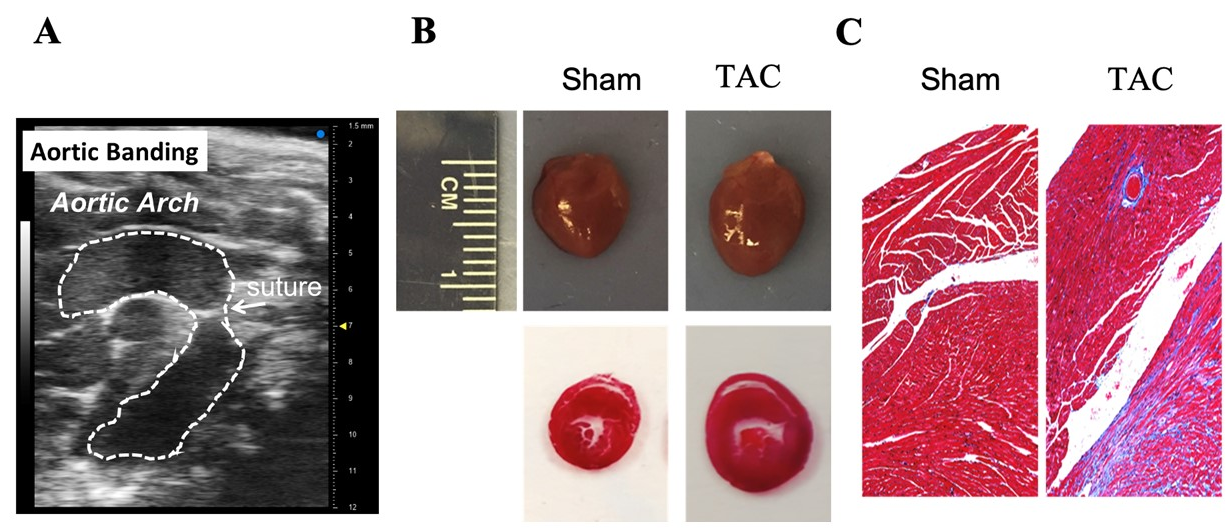


**Supplemental Figure 1.** Validation of mouse model of cardiac hypertrophy by transverse aortic constriction (TAC) by gross pathology and fibrosis. The aortic arch of C57BL/6 mice was constricted between the innominate artery and left common carotid artery for 6 weeks. Sham mice underwent all surgical procedures except constriction of the aorta (sham). **A:** echocardiographic image showing aortic banding in a mouse; **B:** validation of TAC-induced cardiac hypertrophy; **C:** Masson’s trichrome-stained heart sections showing fibrosis elicited by TAC.


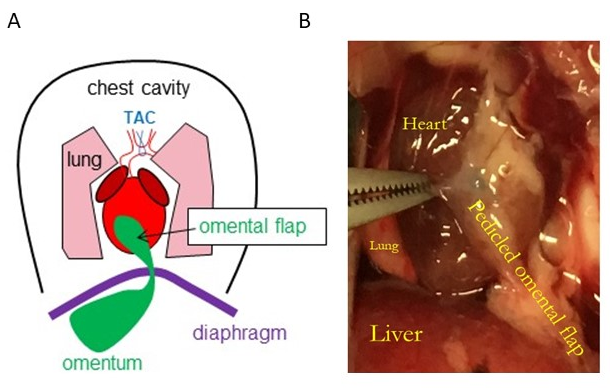


**Supplemental Figure 2.** Transverse aortic constriction (ATC) and cardio-omentopexy. A: Schematic of the cardio-omentopexy procedure after TAC; B: the photograph of cardio-omentopexy in a mouse.


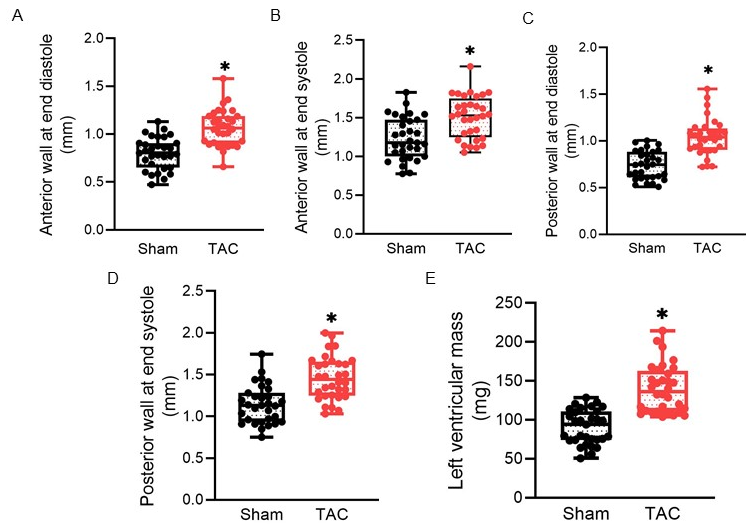


**Supplemental Figure 3.** Left ventricular hypertrophy in C57BL/6 mice undergoing transverse aortic constriction (TAC) for 6 weeks. A: anterior wall at end diastole; B: anterior wall at end systole; B: posterior wall at end diastole; D: posterior wall at end systole; E: left ventricular mass. Sham mice underwent all surgical procedures without constriction of the aorta (sham). Parasternal short axis 2-chamber view-guided M-Mode images of the left ventricle were used to quantitate left ventricular wall thickness and left ventricular mass. The upper and lower borders of the box represent the upper and lower quartiles. The middle horizontal line represents the median. The upper and lower whiskers represent the maximum and minimum values of non-outliers. Larger extra dots represent outliers. P values were determined by 2-way repeated measures analysis of variance followed by post hoc analysis using Mann Whitney test for comparison between 2 groups. *p < 0.05 vs. sham (n = 31-32 mice/group).


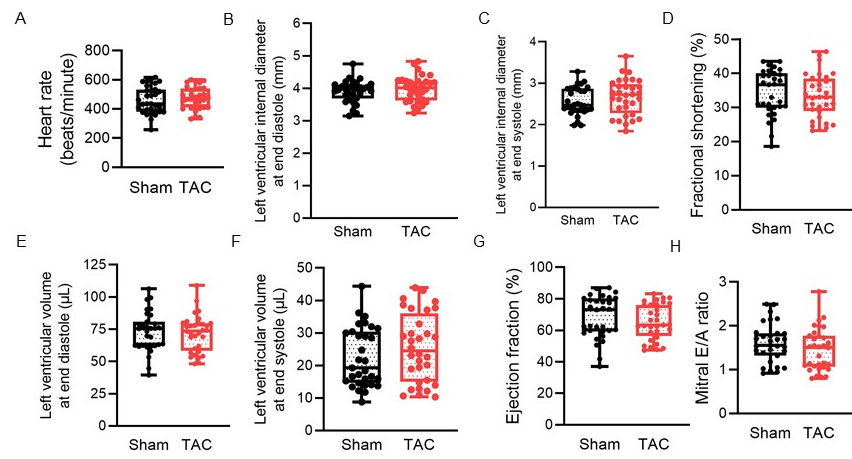


**Supplemental Figure 4.** Echocardiographic parameters of mouse hearts 6 weeks after transverse aortic constriction (TAC). A: heart rate; B: left ventricular internal diameter at end diastole; C: left ventricular internal diameter at end systole: D: fractional shortening: E: left ventricular volume at end diastole: F: left ventricular volume at end systole: G: ejection fraction; F: mitral E/A ratio. The upper and lower borders of the box represent the upper and lower quartiles. The middle horizontal line represents the median. The upper and lower whiskers represent the maximum and minimum values of non-outliers. Extra dots represent outliers. P values were determined by 2-way repeated measures analysis of variance followed by post hoc analysis using Mann Whitney test for comparison between 2 groups. There were no significant differences in all echocardiographic parameters between TAC and sham groups (n = 31-32 mice/group).


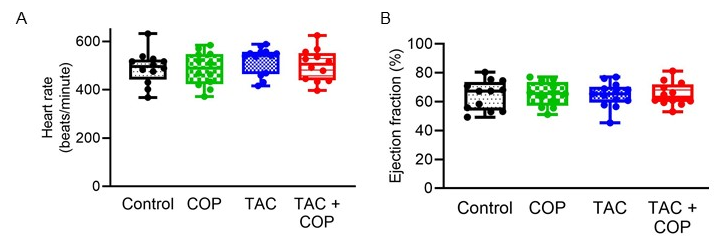


**Supplemental Figure 5.** Heart rate (A) and systolic function (B) of mouse hearts 8 weeks after cardio-omentopexy. Control, the mice were subjected to sham transverse aortic constriction (TAC) surgery for 6 weeks and subsequent sham cardio-omentopexy (COP) surgery for 8 weeks (COP). COP, the mice were subjected to sham TAC surgery for 6 weeks and subsequent COP for 8 weeks. TAC, the mice underwent TAC for 6 weeks and subsequent sham COP surgery for 8 weeks. TAC+COP, the mice were subjected to TAC for 6 weeks and subsequent COP for 8 weeks. The upper and lower borders of the box represent the upper and lower quartiles. The middle horizontal line represents the median. The upper and lower whiskers represent the maximum and minimum values of non-outliers. Extra dots represent outliers. P values were determined by 2-way repeated measures analysis of variance followed by post hoc analysis using Mann Whitney test for comparison between 2 groups. There were no significant differences in heart rate and ejection fraction between groups (n=12-13 mice/group).


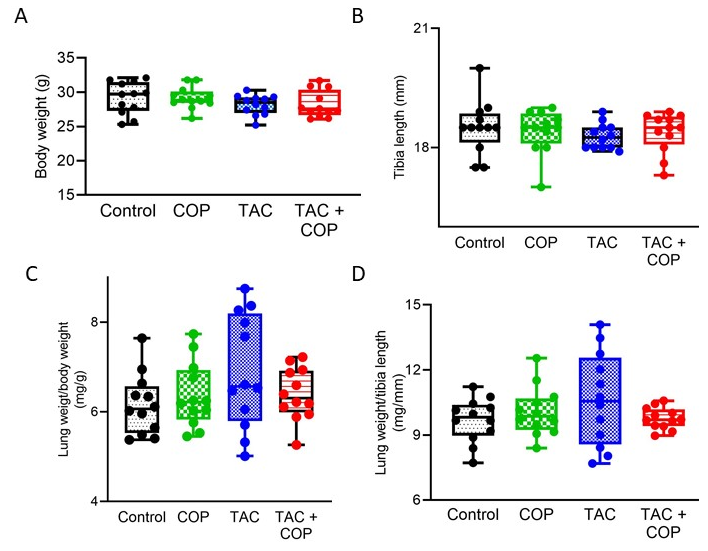


**Supplemental Figure 6.** Physical weights and bone length of experimental groups. Excised lung tissue weight normalized to body weight or tibia length. Control, the mice were subjected to sham TAC surgery for 6 weeks and subsequent sham TAC surgery for 8 weeks (COP). COP, the mice were subjected to sham TAC surgery for 6 weeks and subsequent COP for 8 weeks. TAC, the mice underwent TAC for 6 weeks and subsequent sham COP surgery for 8 weeks. TAC+COP, the mice were subjected to TAC for 6 weeks and subsequent COP for 8 weeks. The upper and lower borders of the box represent the upper and lower quartiles. The middle horizontal line represents the median. The upper and lower whiskers represent the maximum and minimum values of non-outliers. Extra dots represent outliers. P values were determined by 2-way repeated measures analysis of variance followed by post hoc analysis using Mann Whitney test for comparison between 2 groups. There were no significant differences in heart rate and ejection fraction between groups (n=12-13 mice/group).


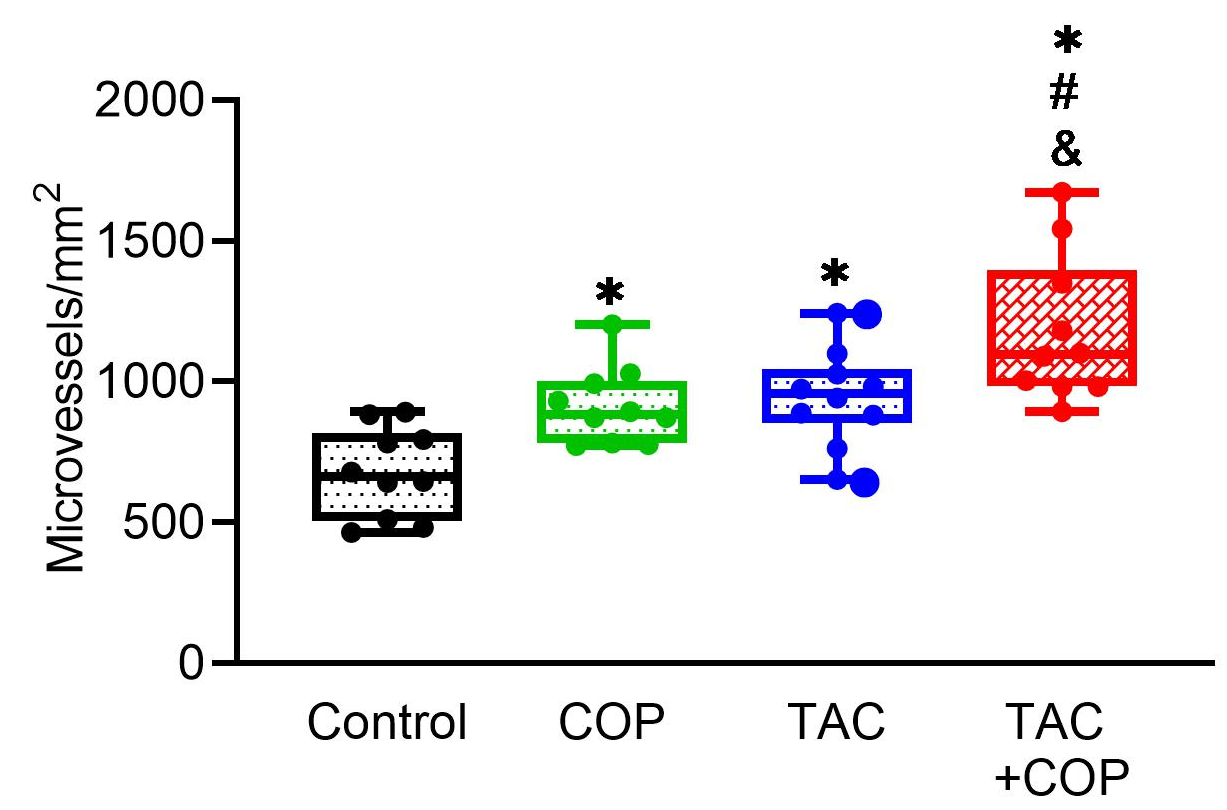


**Supplemental Figure 7.** Quantification of microvessel density in mouse hearts stained with CD31 antibodies. Control, the mice were subjected to sham transverse aortic constriction (TAC) surgery for 6 weeks and subsequent sham cardio-omentopexy (COP) surgery for 8 weeks. COP, the mice were subjected to sham TAC surgery for 6 weeks and subsequent COP for 8 weeks. TAC, the mice underwent TAC for 6 weeks and subsequent sham COP surgery for 8 weeks. TAC+COP, the mice were subjected to TAC for 6 weeks and subsequent COP for 8 weeks. The upper and lower borders of the box represent the upper and lower quartiles. The middle horizontal line represents the median. The upper and lower whiskers represent the maximum and minimum values of non-outliers. Larger extra dots represent outliers. P values were determined by 2-way repeated measures analysis of variance followed by post hoc analysis using Mann Whitney test for comparison between 2 groups. *p < 0.05 versus control; ^#^p < 0.05 versus COP; ^&^P <0.05 versus TAC (n = 10 sections/group).


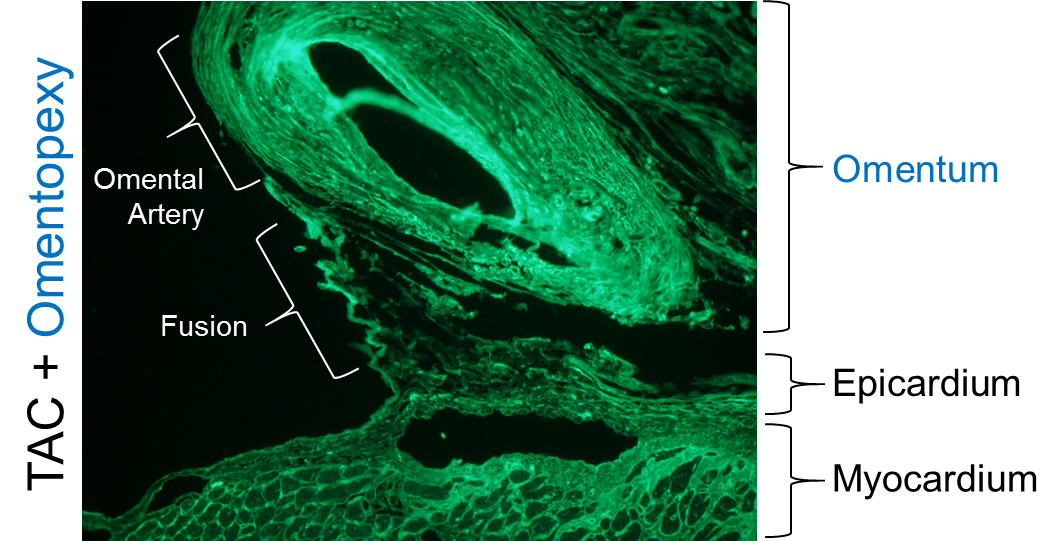

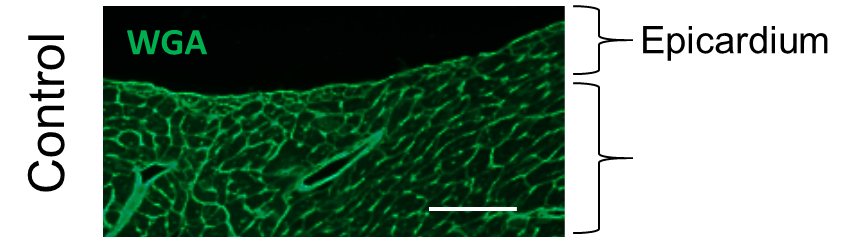


Myocardium

Myocardium

Epicardium

Epicardium

Omentum

TAC + Omentopexy

Control

**Supplemental Figure 8.** Evidence for omental fusion to myocardium in mouse. Displayed are histologic sections of control versus transaortic constricted (TAC) murine hearts followed by cardio-omentopexy. Tissue sections were stained with wheat germ agglutinin/WGA (green), which stains glycoproteins on cell membranes and matrix, to visualize cross cellular sectional area and connective tissue.


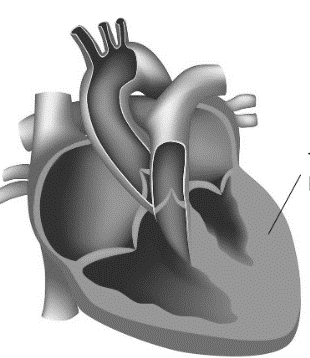


**Supplemental Figure 9.** Candidate pathways by which the omentum may protect the heart after cardio-omentopexy. The omentum may secrete paracrine factors to mobilize cardioprotective endothelial cells (ECs) during angiogenesis. Alternatively, such factors may trigger reparative macrophages (Mɸs) in the heart. It is unclear if such macrophages originate from the omentum, the circulation, or are resident in the heart. Another scenario is that the omentum may mobilize protective progenitor cells (PCs). CM = cardiomyocyte.


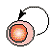


EC

?

Mɸ

PC

**O**

**M**

**E**

**N**

**T**

**U**

**M**

↑*Diastolic*

*Function*

Paracrine

Factors

CM

?

**Supplemental “AHA-11-2021” Video Legend.** Cardio-omentopexy requires a cardioprotective innate immune response to promote myocardial angiogenesis. The video describes the background, hypothesis, methods, results, and conclusions of this study. It will be presented in the American Heart Association Scientific Sessions 2021 in Boston, Massachusetts.

| **SUPPLEMENTAL TABLE 1.** **Echocardiographic parameters of C57BL/6 mice** | | | | |
| --- | --- | --- | --- | --- |
|  | Control  (n = 12) | COP  (n = 12) | TAC  (n = 13) | TAC + COP  (n = 12) |
| *The left ventricle* |  |  |  |  |
| Heart rate (beats/min) | 490±66 | 487±67 | 522±55 | 501±63 |
| Anterior wall at end diastole (mm) | 0.82±0.14 | 0.83±0.12 | 1.22±0.14*^†^ | 1.00±0.17*^&^ |
| Anterior wall at end systole (mm) | 1.24±0.21 | 1.25±0.22 | 1.71±0.18*^†^ | 1.31±0.15*^&^ |
| Posterior wall at end diastole (mm) | 0.86±0.17 | 0.89±0.20 | 1.12±0.10*^†^ | 0.90±0.11*^&^ |
| Posterior wall at end systole (mm) | 1.22±0.18 | 1.24±0.27 | 1.59±0.16*^†^ | 1.30±0.21*^&^ |
| LV internal diameter at end diastole (mm) | 3.93±0.28 | 3.76±0.31 | 3.79±0.31 | 3.97±0.36 |
| LV internal diameter at end systole (mm) | 2.64±0.33 | 2.51±0.24 | 2.58±0.42 | 2.66±0.33 |
| Fractional shortening (%) | 33±6 | 33±4 | 32±7 | 33±4 |
| LV end-diastolic volume (µL) | 68±15 | 62±7 | 61±11 | 69±14 |
| LV end-systolic volume (µL) | 24±9 | 21±5 | 22±8 | 25±10 |
| Ejection fraction (%) | 64±10 | 65±8 | 65±8 | 65±8 |
| LV mass (mg) | 96±6 | 96±18 | 148±21*^†^ | 114±15*^&^ |
| Cardiac output (mL/min) | 19±5 | 18±3 | 20±4 | 21±3 |
| Mitral E/A ratio | 1.64±0.31 | 1.75±0.29 | 1.21±0.26*^†^ | 1.63±0.32 |
| *Aorta* |  |  |  |  |
| Peak velocity of aortic valve (m/s) | 1.27±0.26 | 1.24±0.20 | 1.15±0.23 | 1.19±0.26 |
| Transverse aortic lumen diameter (mm) | 1.39±0.32 | 1.39±0.24 | 1.77±0.32*^†^ | 1.69±0.25*^†^ |
| TAC pressure gradient (mmHg) | 2.8±0.8 | 2.7±0.5 | 59.5±30.7*^†^ | 57.1±28.6*^†^ |

Control, the mice were subjected to sham transverse aortic constriction (TAC) surgery for 6 weeks and subsequent sham cardio-omentopexy (COP) surgery for 8 weeks (COP). COP, the mice were subjected to sham TAC surgery for 6 weeks and subsequent COP for 8 weeks. TAC, the mice underwent TAC for 6 weeks and subsequent sham COP surgery for 8 weeks. TAC+COP, the mice were subjected to TAC for 6 weeks and subsequent COP for 8 weeks. Mean and SD. Kruskal-Wallis test followed by Dunn’s test was used to analyze multiple group comparisons. *indicates p<0.05 versus control, ^†^indicates p<0.05 versus COP, ^&^indicates p<0.05 versus TAC (n=12-13 mice/group).

| **SUPPLEMENTAL TABLE 2.** **Echocardiographic and physical parameters of C57BL/6 mice receiving clodronate-liposome treatment** | | |
| --- | --- | --- |
|  | TAC+COP  +PBS  (n = 8) | TAC+COP  +Clodronate  (n = 8) |
| Heart rate (beats/min) | 483±61 | 496±84 |
| Anterior wall at end diastole (mm) | 0.97±0.15 | 1.13±0.11* |
| Anterior wall at end systole (mm) | 1.34±0.11 | 1.52±0.14 |
| Posterior wall at end diastole (mm) | 0.91±0.10 | 1.12±0.17* |
| Posterior wall at end systole (mm) | 1.20±0.16 | 1.56±0.34* |
| Left ventricular internal diameter at end diastole (mm) | 4.27±0.31 | 4.38±0.39 |
| Left ventricular internal diameter at end systole (mm) | 2.90±0.26 | 3.11±0.47 |
| Fractional shortening (%) | 32±3 | 29±7 |
| Left ventricular end-diastolic volume (µL) | 83±13 | 91±14 |
| Left ventricular end-systolic volume (µL) | 31±8 | 38±17 |
| Ejection fraction (%) | 63±6 | 59±15 |
| Left ventricular mass (mg) | 120±14 | 168±37* |
| Cardiac output (mL/min) | 23±3 | 26±6 |
| Mitral E/A ratio | 1.59±0.28 | 1.23±0.19* |
| Heart weight (mg) | 162±12 | 208±15* |
| Heart weight/body weight (mg/g) | 5.9±0.3 | 6.8±0.3* |
| Heart weight/tibia length (mg/mm) | 9.9±0.63 | 11.5±0.98* |
| Left ventricle weight/body weight (mg/g) | 4.3±0.4 | 5.0±0.4* |

The mice in TAC+COP+PBS group were subjected to transverse aortic constriction (TAC) for 6 weeks and subsequent cardio-omentopexy (COP) for 8 weeks and given phosphate buffered saline (PBS) during 8 weeks of COP. The animals in TAC+COP+Clodro group were subjected to TAC for 6 weeks and subsequent COP for 8 weeks and injected clodronate-liposome (Clodro) during 8 weeks of COP. Mean and SD. P values were determined by 2-way repeated measures analysis of variance followed by post hoc analysis using Kruskal-Wallis test for comparison between 2 groups. *indicates p < 0.05 versus TAC+COP+PBS (n = 8 mice/group).

**References**

11. Wilkosz S, Ireland G, Khwaja N, et al. A comparative study of the structure of human and murine greater omentum. *Anat Embryol.* 2005;209(3):251-61.

12. Zaw AM, Williams CM, Law HK, Chow BK. Minimally invasive transverse aortic constriction in mice. *J Vis Exp.* 2017(121).

13. Pant T, Dhanasekaran A, Zhao M, et al. Identification and analysis of circulating long non-coding RNAs with high significance in diabetic cardiomyopathy. *Sci Rep.* 2021;11(1):2571.

14. Ge ZD, Li Y, Qiao S, et al. Failure of isoflurane cardiac preconditioning in obese type 2 diabetic mice involves aberrant regulation of microRNA-21, endothelial nitric-oxide synthase, and mitochondrial complex I. *Anesthesiology.* 2018;128(1):117-29.

15. Liebermann-Meffert D. The greater omentum. anatomy, embryology, and surgical applications. *Surg Clin North Am.* 2000;80(1):275-293, xii.

16. Ge ZD, Ionova IA, Vladic N, et al. Cardiac-specific overexpression of GTP cyclohydrolase 1 restores ischaemic preconditioning during hyperglycaemia. *Cardiovasc Res.* 2011;91(2):340-9.

17. Baumgardt SL, Paterson M, Leucker TM, et al. Chronic co-administration of sepiapterin and L-citrulline ameliorates diabetic cardiomyopathy and myocardial ischemia/reperfusion injury in obese type 2 diabetic mice. *Circ Heart Fail.* 2016;9(1):e002424.

18. Wu HE, Baumgardt SL, Fang J, et al. Cardiomyocyte GTP cyclohydrolase 1 protects the heart against diabetic cardiomyopathy. *Sci Rep.* 2016;6:27925.

19. Boehm M, Tian X, Mao Y, et al. Delineating the molecular and histological events that govern right ventricular recovery using a novel mouse model of pulmonary artery de-banding. *Cardiovasc Res.* 2020;116(10):1700-9.

20. DeBerge M, Yeap XY, Dehn S, et al. MerTK cleavage on resident cardiac macrophages compromises repair after myocardial ischemia reperfusion injury. *Circ Res.* 2017;121(8):930-40.

21. Dick SA, Macklin JA, Nejat S, et al. Self-renewing resident cardiac macrophages limit adverse remodeling following myocardial infarction. *Nat Immunol.* 2019;20(1):29-39.

22. Peet C, Ivetic A, Bromage DI, Shah AM. Cardiac monocytes and macrophages after myocardial infarction. *Cardiovasc Res.* 2020;116(6):1101-12.

23. Moreno SG. Depleting macrophages in vivo with clodronate-liposomes. *Methods Mol Biol.* 2018;1784:259-62.
