# Supplementary figures and images for: Cardio-omentopexy requires a cardioprotective innate immune response to promote myocardial angiogenesis in mice
Source: JTCVS Open. 2022 Feb 24;10:222–42. doi: 10.1016/j.xjon.2022.02.027 (PMC9390370; doi:10.1016/j.xjon.2022.02.027)

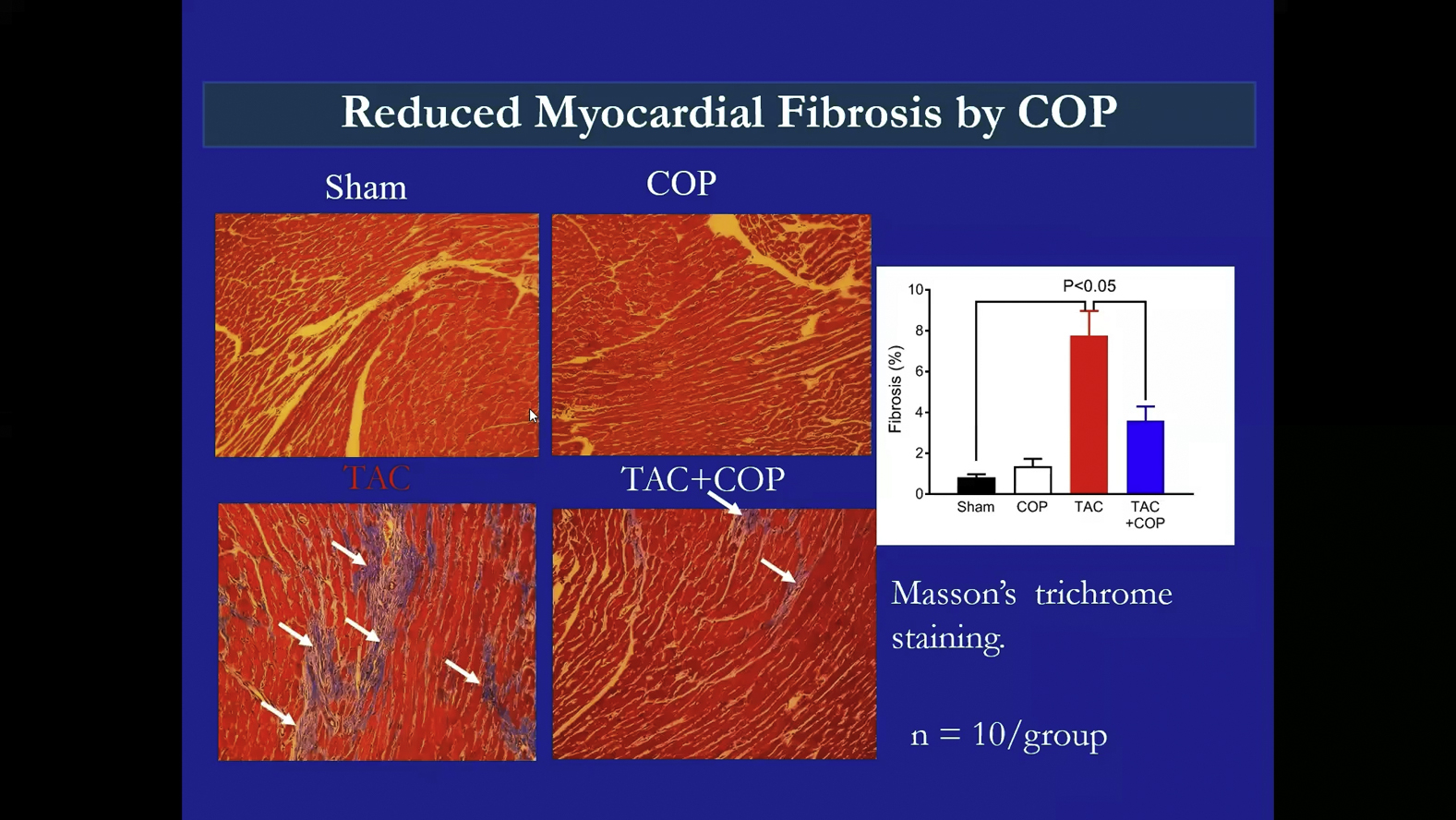

Supplement: Video 1 — Cardio-omentopexy requires a cardioprotective innate immune response to promote myocardial angiogenesis. The video describes the background, hypothesis, methods, results, and conclusions of this study. It will be presented in the American Heart Association Scientific Sessions 2021 in Boston, Massachusetts. Video available at: https://www.jtcvs.org/article/S2666-2736(22)00096-1/fulltext. [file fx3.jpg]
